# Direct Cytosolic Delivery of Proteins and CRISPR-Cas9 Genome Editing by Gemini Amphiphiles via Non-Endocytic Translocation Pathways

**DOI:** 10.1021/acscentsci.3c00207

**Published:** 2023-06-08

**Authors:** Zhicheng Le, Qi Pan, Zepeng He, Hong Liu, Yi Shi, Lixin Liu, Zhijia Liu, Yuan Ping, Yongming Chen

**Affiliations:** † School of Materials Science and Engineering, Key Laboratory for Polymeric Composite and Functional Materials of Ministry of Education, Sun Yat-sen University, Guangzhou 510006, China; ‡ College of Pharmaceutical Sciences, Zhejiang University, Hangzhou 310058, China

## Abstract

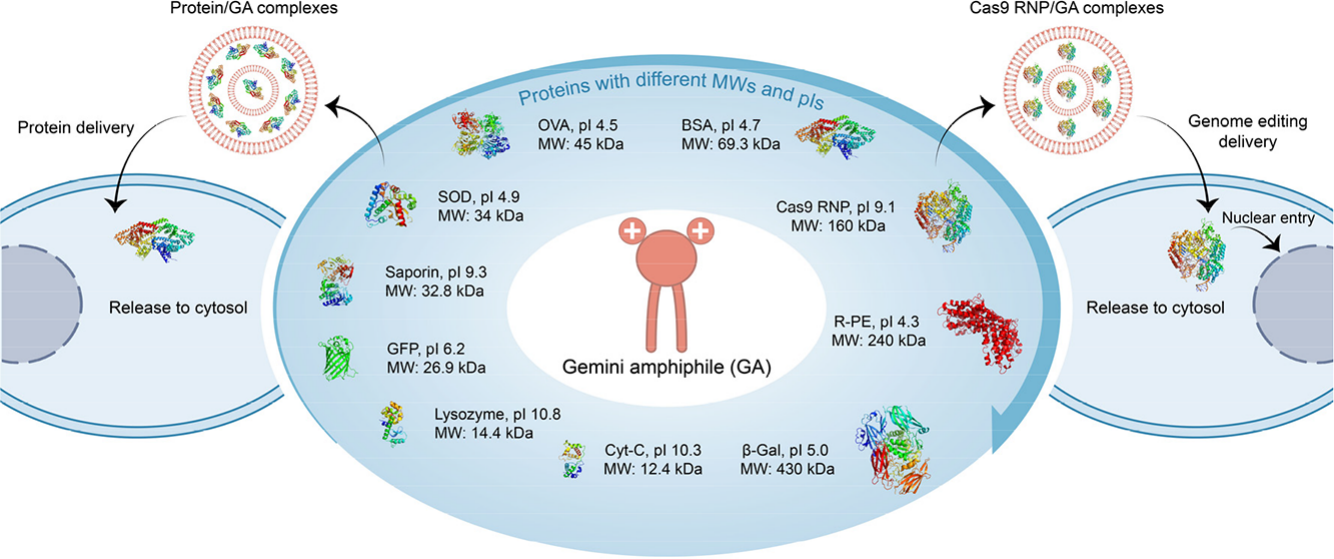

Intracellular delivery of therapeutic biomacromolecules is often challenged by the poor transmembrane and limited endosomal escape. Here, we establish a combinatorial library composed of 150 molecular weight-defined gemini amphiphiles (GAs) to identify the vehicles that facilitate robust cytosolic delivery of proteins in vitro and in vivo. These GAs display similar skeletal structures but differential amphiphilicity by adjusting the length of alkyl tails, type of ionizable cationic heads, and hydrophobicity or hydrophilicity of a spacer. The top candidate is highly efficient in translocating a broad spectrum of proteins with various molecular weights and isoelectric points into the cytosol. Particularly, we notice that the entry mechanism is predominantly mediated via the lipid raft-dependent membrane fusion, bypassing the classical endocytic pathway that limits the cytosolic delivery efficiency of many presently available carriers. Remarkably, the top GA candidate is capable of delivering hard-to-deliver Cas9 ribonucleoprotein in vivo, disrupting *KRAS* mutation in the tumor-bearing mice to inhibit tumor growth and extend their survival. Our study reveals a GA-based small-molecule carrier platform for the direct cytosolic delivery of various types of proteins for therapeutic purposes.

## Abstract 2

A robust gemini amphiphile-based carrier platform for facilitating direct cytosolic delivery of broad-spectrum proteins, including Cas9 ribonucleoproteins, through the lipid raft-dependent membrane fusion mechanism.

## 1 Introduction

Intracellular delivery of exogenous biomacromolecules is of wide interest in biology and medicine. Particularly, the cytosolic delivery of therapeutic biomacromolecules, such as proteins and nucleic acids, shows great potential for treating a broad array of disorders associated with intracellular targets.^1−5^ Nevertheless, the impermeable cell membrane and/or the endosomal entrapment constitutes the formidable barriers limiting the efficient entry of these biomacromolecules into the cytosol, and thus, the development of safe and efficient strategies that can translocate therapeutic biomacromolecules into cells in its functionally active form is of substantial importance.^6−9^ Currently, effective approaches to surmount these delivery barriers are heavily dependent on carrier systems, such as inorganic nanoparticles,^10^ synthetic polymers,^11−17^ peptides,^18−20^ and even microscaled coacervates,^21^ to directly cross the membrane, or mediate endosomal escape following the endocytosis. Alternatively, these biomacromolecules are covalently conjugated with delivery carriers, such as dynamic polyconjugates (DPCs) and triantennary *N*-acetylgalactosamine (GalNAc) conjugates,^22^ to access the cytosol. Although these strategies are generally effective and increasingly considered for clinical translation, they also suffer from a few shortcomings. First, carrier preparation and manufacturing can be complex, laborious and expensive, and the precise control of their properties remains elusive. Second, most of the carriers are restricted to delivering particular types of cargoes, thus greatly narrowing down their application scope for other macromolecular therapeutics. Third, inefficient delivery, especially for carrier systems that are internalized via the endocytic pathway (about 1% endosomal escape), limits the wide utility of carrier-based approaches.^1,23^ Thus, the development of a robust and universal carrier system with well-defined features may provide new opportunities to broaden the scope of macromolecular therapeutics.

Recent developments in delivery systems for biomacromolecules have shed light on the rational design of carriers. For example, as proteins usually possess dramatically different three-dimensional structures and molecular weights (MWs), isoelectric points (pIs), surface charge density and the distribution of hydrophobic/hydrophilic domains, suitable molecular interactions, such as hydrophobic forces and electrostatic interactions between cargoes and carriers, are essential for the effective complexation of proteins.^15^ In addition, the carriers chemically encoded with ionizable structures or membrane disruptive domains would promote the endosomal escape of the entrapped biomacromolecules.^24−27^ These requirements enable the polymers as the most promising candidate in that they are easy to be fulfilled with functional properties overcoming the delivery barriers by encoding desirable chemical structures into different monomers, main or side chains. Despite these merits, polymers still suffer from several drawbacks impeding their clinical development as protein delivery carriers. Particularly, their polydispersity nature, hard-to-control molecular weights, and batch-to-batch variations make polymeric carriers particularly elusive for quality control and clinical translation. In contrast, small molecules with well-defined molecular weights and structures are much easier to be translated; however, the utilization of small molecules to deliver a broad spectrum of proteins has yet to become commonplace. Only a few small molecule-based, multicomponent systems, such as cationic liposomes and lipid nanoparticles, have been developed as the delivery carriers for customized anionic proteins through electrostatic interactions, but they are difficult to deliver large native proteins or their complexes and are usually accompanied by cytotoxicity issues.^28,29^

**Figure 1:**
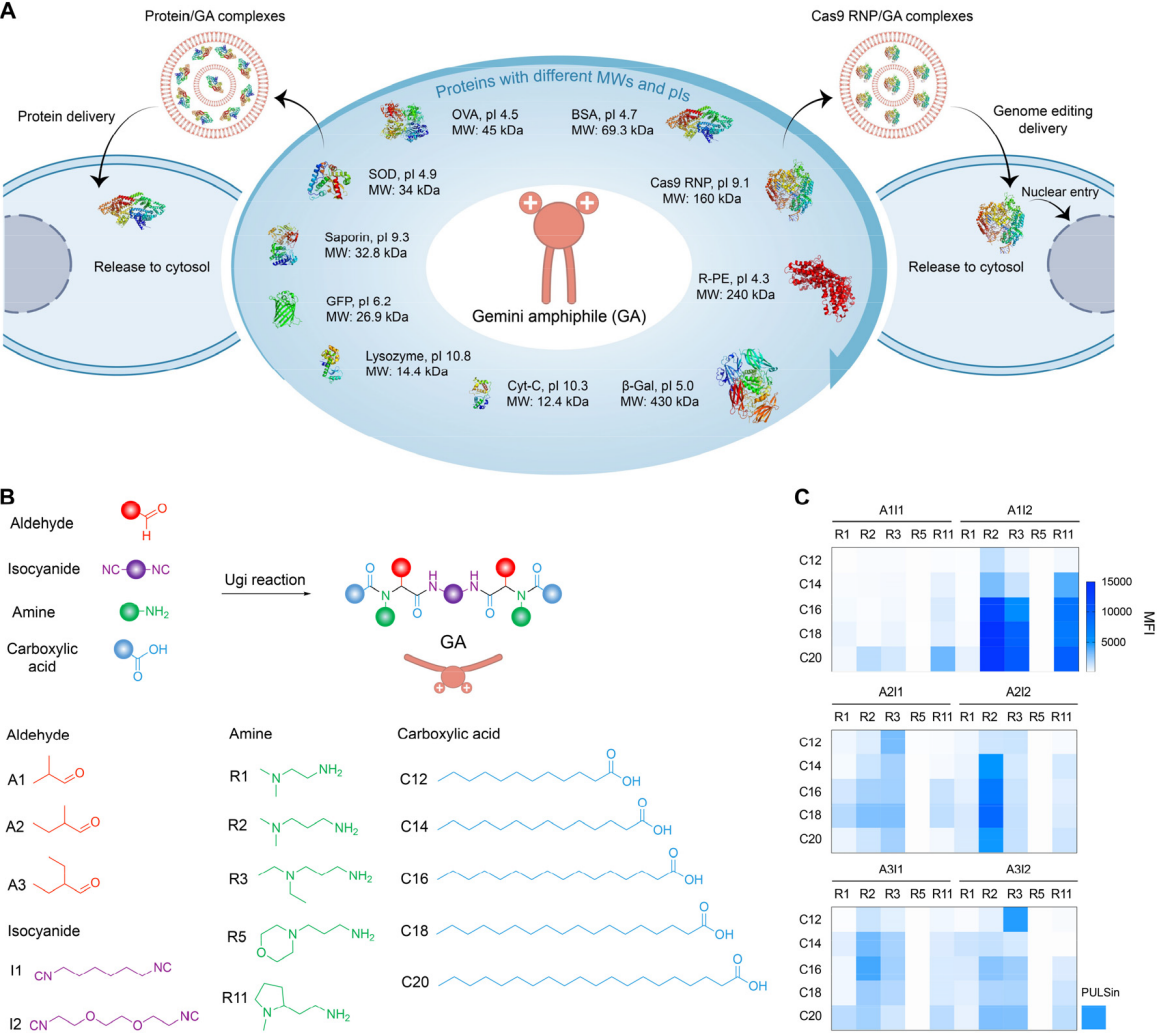
Combinational synthesis of GAs for intracellular protein delivery. A) Schematic of cytosolic delivery of various proteins and Cas9 RNP by the GAs. B) Incorporation of aldehydes (A), diisocyanides (I), amines (R), and carboxylic acids (C) into GAs via one-step Ugi multicomponent reaction and layout of the combinational approach. C) Heat maps of mean fluorescence intensity (MFI) after HeLa cells treated with BSA-FITC/GA complexes at their corresponding optimal conditions for 4 h. BSA-FITC was kept at 4 μg/mL. PULSin was used as a positive control (*n* = 3).

In view of the above challenges, we designed a molecular library of gemini amphiphiles (GAs) with tunable charges and hydrophobic properties for the cytosolic delivery of proteins. GAs are typically composed of two identical amphiphilic units linked by an organic spacer, and they usually exhibit superior performances for biomedical applications owing to their unique solution features, such as enhanced surfactant property, variable aggregate structure and lower level of toxicity.^30−32^ Herein, such a symmetrical structure can be easily synthesized by a one-step, Ugi four-component reaction to quickly establish the carrier library. These GAs share structural similarities with symmetrical hydrophobic tails and ionizable cationic heads linked by hydrophilic or hydrophobic spacers. By carefully screening the type of tertiary-amine-containing heads, length of hydrophobic tails, as well as hydrophilicity/hydrophobicity of space linkers, we obtained more than 150 candidates and identified GAs with saturated alky tails of 18–20 carbon atoms, suitable ionizable heads and hydrophilic space linkers could efficiently mediate intracellular delivery of a wide range of proteins with various MWs or pIs, including Cas9 ribonucleoproteins (RNP), into the cytoplasm (Figure 1A). Interestingly, we show that the GAs bypass classical endocytic pathways to directly translocate protein cargos into cells by means of the lipid raft-dependent membrane fusion mechanism. These exciting and promising outcomes define an alternative, unconventional generation of intracellular delivery vectors for proteins, opening an avenue for the rational design of a well-defined carrier platform for the direct cytosolic delivery of macromolecular therapeutics.

## 2 Results

### Molecular Library Construction and Biological Screening of GAs

Here, a molecular library containing 150 GAs, which were combinatorially synthesized via the isocyanide-mediated Ugi four-component reaction, was established. More specifically, diisocyanides (I1 and I2) acted as the hydrophilic or hydrophobic spacers to connect two identical amphiphilic units with symmetrical hydrophobic tails and ionizable cationic heads in the reaction. Five kinds of amines (R1, R2, R3, R5 and R11) were adopted to tune the charge property of GAs, and three α-substituted aldehydes (A1, A2 and A3) and five carboxylic acids (C12, C14, C16, C18 and C20) with various alkyl tails were chosen to control the overall hydrophobicity of GAs. These synthetic GAs were termed as AaIbRxCy, where Aa, Ib, Rx and Cy stand for the reacted aldehydes (a = 1, 2 and 3), diisocyanides (b = 1 and 2), amines (x = 1, 2, 3, 5 and 11) and carboxylic acids (y = 12, 14, 16, 18 and 20), respectively (Figure 1B). To identify the candidates with ability to mediate intracellular protein delivery from the library of 150 GAs, fluorescein isothiocyanate (FITC)-labeled bovine serum albumin (BSA-FITC), was used as the model protein to complex with GAs at various mass ratios to form BSA-FITC/GA complexes, then the intracellular mean fluorescence intensity (MFI) of HeLa cells was quantified by the flow cytometry after various BSA-FITC/GA complexes incubated with HeLa cells for 4 h (Figure S1). From the in vitro screening heat map, we found 14 GAs dramatically improved intracellular delivery efficiency in comparison with PULSin (a commercial protein delivery reagent) at their corresponding optimal conditions (Figure 1C). Therefore, Ugi-based multicomponent reaction offers an efficient and combinatorial approach to construct the molecular library, allowing the rapid and facile synthesis of GA candidates for large-scale screening.

### Screening of GA Carriers and Optimization of Cytosolic Delivery

**Figure 2:**
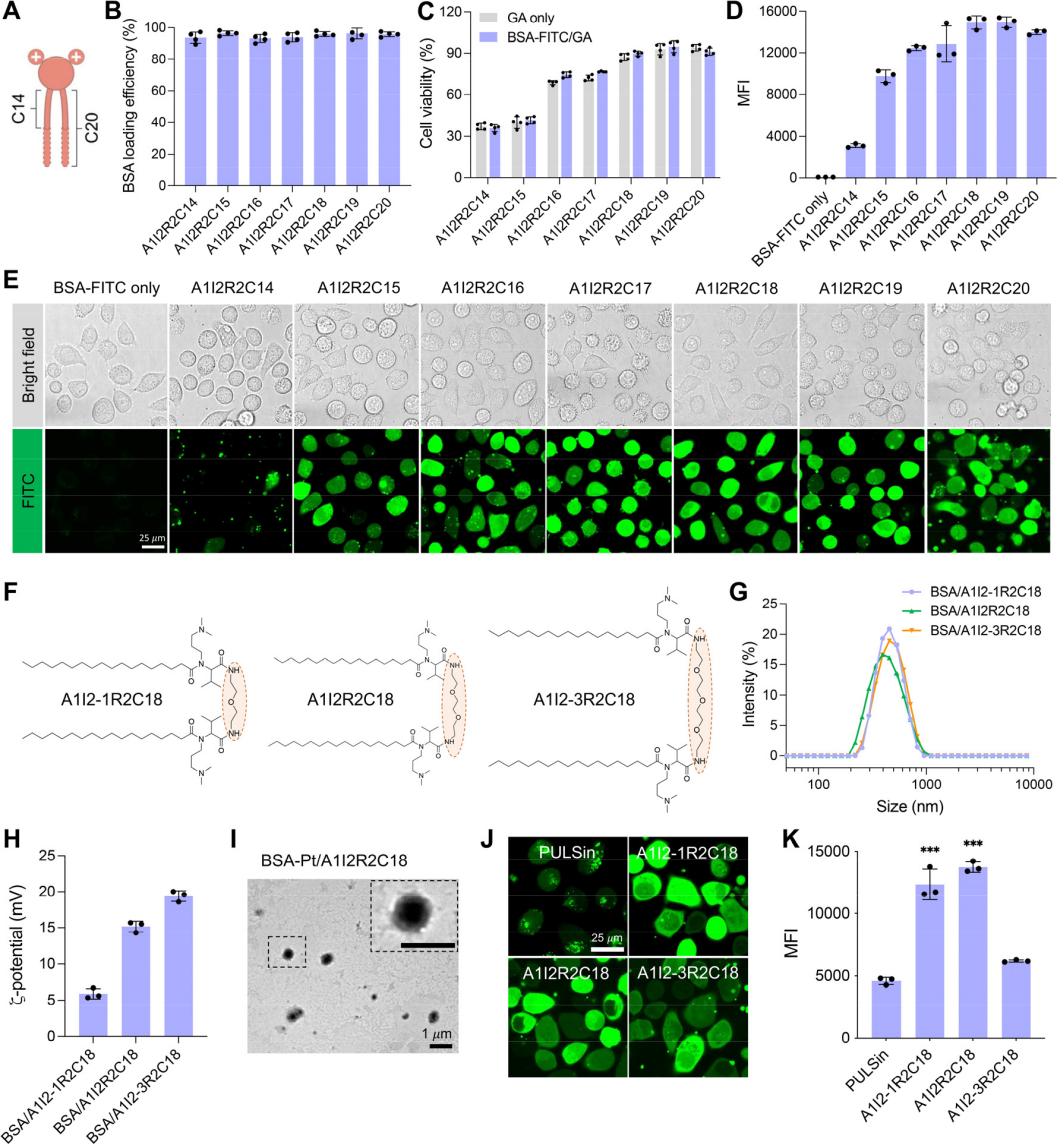
Structure optimization of GAs for improving cytosolic protein delivery. A) Structural illustration of GAs with symmetrical alkyl chain tails containing carbon atom numbers from 14 to 20. B) Protein loading efficiency of BSA/GA complexes as determined by the Pierce BCA Protein Assay Kit. BSA was 4 μg/mL and GAs were 8 μg/mL. (*n* = 4). C) Cell viability of HeLa cells after treatment with various GAs (A1I2R2C14-A1I2R2C20) or their corresponding BSA-FITC/GA complexes. BSA-FITC and GAs were kept at 4 and 8 μg/mL, respectively. (*n* = 4). D) MFI of HeLa cells treated with various BSA-FITC/GA complexes. BSA-FITC only was set as a negative control. BSA-FITC and GAs were kept at 4 μg/mL and 8 μg/mL, respectively. (*n* = 3). E) Representative images of HeLa cells treated with BSA-FITC only or various BSA-FITC/GA complexes. BSA-FITC was 4 μg/mL and GAs were 8 μg/mL. Scale bar is 25 μm. F–H) Chemical structures of GAs with a single ether bond spacer (A1I2-1R2C18), two ether bonds spacer (A1I2R2C18) or three ether bonds spacer (A1I2-3R2C18) (F), and their corresponding size distribution (G) and ζ-potentials (H) after being complexed with BSA. BSA was 4 μg/mL and GAs were 8 μg/mL (*n* = 3). I) Particle morphology of complexes as observed by transmission electron microscopy (TEM). BSA was labeled with Pt (BSA-Pt), scale bar is 1 μm. J and K) Intracellular fluorescence distribution (J) and MFI (K) after HeLa cells treated with BSA-FITC/PULSin, or BSA-FITC/A1I2-1R2C18, BSA-FITC/A1I2R2C18 and BSA-FITC/A1I2-3R2C18 complexes, respectively. BSA-FITC was 4 μg/mL and GAs were 8 μg/mL. ***P < 0.001 of A1I2-1R2C18 and A1I2R2C18 compared to PULSin or A1I2-3R2C18 (*n* = 3). Scale bar is 25 μm.

To understand how delicate structure impacts its intracellular delivery performance, we carefully varied each functional moiety of GAs, including aldehydes, diisocyanides, amines, and carboxylic acids. First, the GA that could mediate intracellular BSA-FITC delivery to yield higher intracellular MFI as compared with the PULSin was defined as the hit GA. We found that the hit number of GAs obviously decreased in the aldehydes with increased methylene of alkyl chains, and isobutyraldehyde (A1), the α-substitution aldehyde with minimum methylene, showed the highest hit number of GAs (Figure S2A). Compared to other amines, we observed that R2 was the optimal amine structure for producing GAs with the highest hit numbers (Figure S2B). Additionally, the increase in alkyl chain length of carboxylic acids from C12 to C20 appeared to increase the relative hit number of GAs; however, the trend reaches a plateau when the chain length varied from C16 to C20 (Figure S2C). To further investigate whether GAs with even or odd alkyl chain length would affect the intracellular protein delivery performance, we systemically compared intracellular BSA-FITC delivery ability mediated by these GAs with the successive increase of alkyl carbon atoms from C14 to C20 (Figure 2A). All GAs with alkyl chain length from C14 to C20 (A1I2R2C14–A1I2R2C20) could efficiently bind to BSA to form complexes with loading efficiency higher than 90% (Figure 2B), and these BSA/GA complexes showed similar particle size range (ca. 400–600 nm) with slightly positive surface charges (5–15 mV) (Figure S3). It is worth noting that GAs with a shorter alkyl chain tail (A1I2R2C14 and A1I2R2C15) generated remarkable cytotoxicity with relative cell viability less than 50%, but the cytocompatibility could be greatly improved by increasing the alkyl chain length of GAs from C16 to C20 due to the higher 50% inhibitory concentration (IC50) values (Figure 2C and Figure S4). Of note, A1I2R2C18 and A1I2R2C19 are the most efficient in delivering protein intracellularly, with FITC-positive rates of about 94% and 89%, respectively (Figure 2D and Figure S5). Whereas HeLa cells incubated with BSA-FITC hardly showed any fluorescence signals, the cells incubated with various BSA-FITC/GA complexes yielded bright green fluorescence in the cytoplasm except for A1I2R2C14 (Figure 2E). Collectively, these results indicated that GAs with alkyl chain lengths from C15 to C20 (A1I2R2C15–A1I2R2C20) are capable of transporting proteins into cells.

Next, we investigate how diisocyanide, the space linker, impacts the intracellular protein delivery of GAs. We first noted hydrophilic diisocyanides containing ether bonds (I2) displayed a much higher hit number as compared with hydrophobic alkyl chain spacer (I1) (Figure S2D). Based on the above results, we evaluated the effect of ether bond number of diisocyanides on delivery efficiency (Figure 2F and Figures S6–S8). The formed three BSA/GA complexes, including BSA/A1I2-1R2C18 (with one ether bond), BSA/A1I2R2C18 (with two ether bonds) and BSA/A1I2-3R2C18 (with three ether bonds), showed similar size distribution (ca. 500 nm), positively charged surfaces as well as high protein loading efficiency (>90%) (Figure 2G,H and Figure S9). To determine the particle morphology of BSA/GA complexes, Pt-labeled BSA (BSA-Pt) was used to enhance the contrast under the transmission electron microscopy (TEM) observation (Figure S10). The TEM image indicated that the complex BSA-Pt/A1I2R2C18 exhibited spherical structure and negatively charged BSA was mainly trapped into the inner core of complexes (Figure 2I), which was indirectly verified by the positive surface charges as mentioned above. Furthermore, confocal laser scanning microscope (CLSM) images revealed that BSA-FITC can be efficiently delivered into the cytoplasm by three GAs, as indicated by strong green fluorescence (Figure 2J). It is worth noting that both A1I2-1R2C18- and A1I2R2C18-mediated BSA-FITC delivery showed comparable intracellular MFI, which was significantly higher than the treatment by either A1I2-3R2C18 or the commercial protein delivery reagent (PULSin) (Figure 2K). It was reported that a longer hydrophilic spacer can increase the overall hydrophilicity of GAs.^33^ Herein, we measured the critical aggregation concentration (CAC) of A1I2-3R2C18 with 3.5- and 3.3-fold higher than A1I2-1R2C18 and A1I2R2C18, respectively (Figure S11), which suggested that the decreased hydrophobicity of A1I2-3R2C18 likely leads to the negative effect on its affinity to lipophilic cell membranes and therefore decrease intracellular protein delivery efficiency. Besides, the BSA-FITC delivered by the representative A1I2R2C18 showed increased fluorescence intensity in a protein dose-dependent manner (Figure S12), and the fluorescence intensity of transfected HeLa cells has no change before and after treatment of trypan blue, which is a membrane-impermeable dye to quench the fluorescence of BSA-FITC absorbed on cell membranes,^34^ suggesting that the delivered BSA-FITC was almost distributed inside cells (Figure S13). These findings indicate that diisocyanide space linkers with suitable hydrophilic ether bonds contribute to GA-mediated intracellular delivery of proteins.

### Cellular Internalization by the Nonendocytic Translocation Pathways

**Figure 3:**
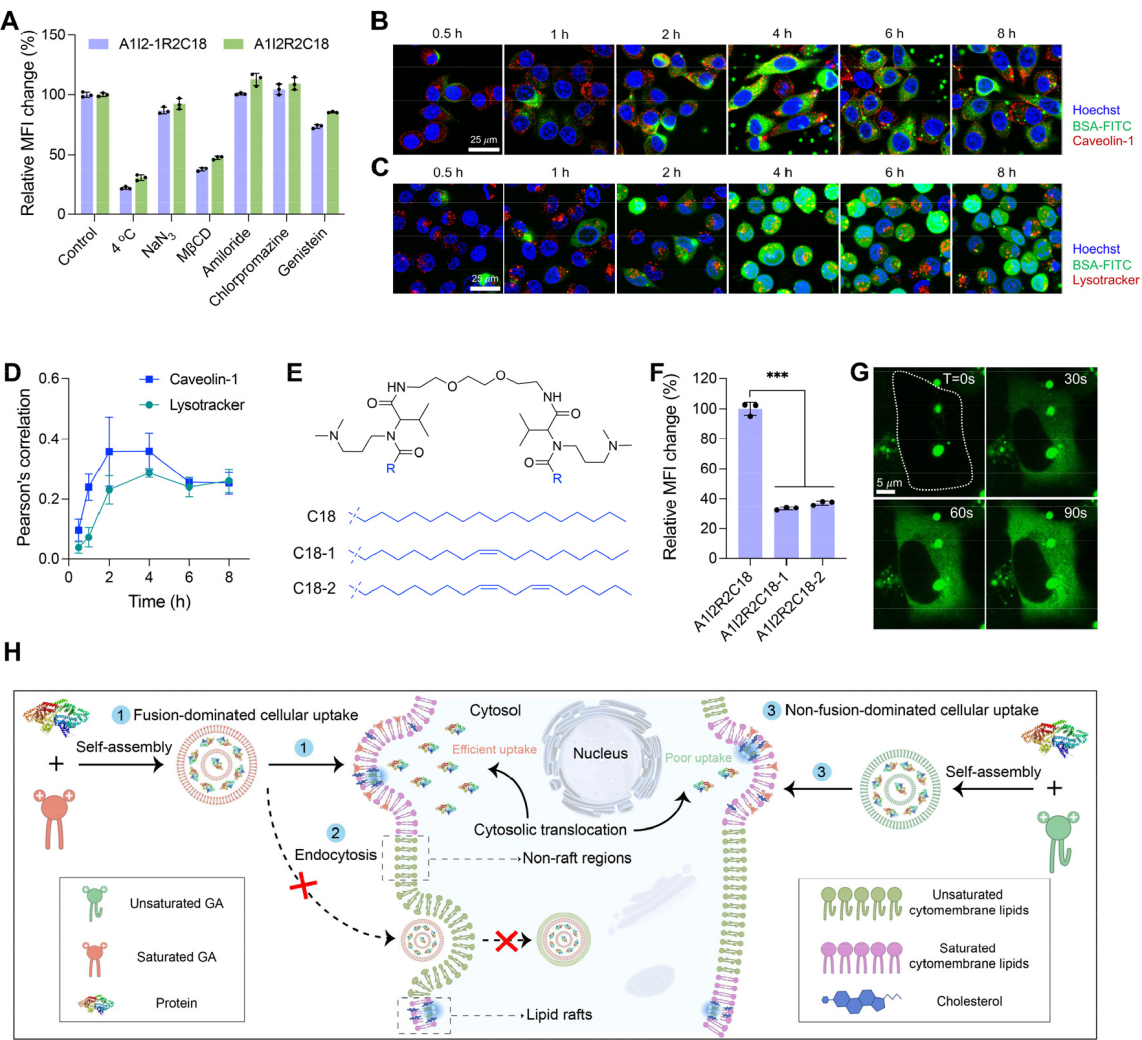
Internalization mechanism study. A) Relative MFI change after HeLa cells pretreated with various endocytic inhibitors, followed by incubating with BSA-FITC/A1I2-1R2C18 or BSA-FITC/A1I2R2C18 complexes for 4 h. BSA-FITC was 4 μg/mL, and GAs were 8 μg/mL. (*n* = 3). B–D) Confocal microscopy images (B and C) and their corresponding Pearson’s correlation coefficients (D) of BSA-FITC with caveolin or endo/lysosomes (*n* = 3) after HeLa cells treated with the representative BSA-FITC/A1I2R2C18 complexes at various time intervals. The nucleus was stained with Hoechst (blue), and caveolin or endo/lysosomes were stained with anticaveolin-1 antibody and lysotracker red (red), respectively. BSA-FITC was 4 μg/mL and A1I2R2C18 was 8 μg/mL. Scale bar is 25 μm. E–F) Intracellular delivery of BSA-FITC mediated by GAs with different degrees of saturation, including A1I2R2C18, oleic acid (C18-1)-derived GA (A1I2R2C18-1) and linoleic acid (C18-2)-derived GA (A1I2R2C18-2) (E), and the relative MFI change (F) after incubation of BSA-FITC/GA complexes with HeLa cells for 4 h. BSA-FITC was 4 μg/mL. Each GA was at the optimal concentration, namely, A1I2R2C18 was 8 μg/mL, A1I2R2C18-1 was 4 μg/mL and A1I2R2C18-2 was 2 μg/mL. ***P < 0.001 (*n* = 3). G) Time-lapse imaging of BSA-FITC after 1 h incubation of HeLa cells with BSA-FITC/A1I2R2C18 complexes, *T* = 0 s represented the start time of the delivery event. BSA-FITC was 4 μg/mL and A1I2R2C18 was 8 μg/mL. Scale bar is 5 μm. H) The illustration of our proposed intracellular delivery mechanism mediated by the GAs. On incubation with cells, the preferential interactions between saturated hydrophobic tails of GAs and lipid raft domains would improve the direct cytosolic delivery of biomacromolecules via the lipid raft-dependent membrane fusion mechanism, thus bypassing the classical endocytic pathway.

Next, we investigated the cellular internalization mechanism mediated by the two top-performing GAs by pretreating HeLa cells with various endocytic inhibitors. As shown in Figure 3A, we observed that none of the two inhibitors, including amiloride (the macropinocytosis inhibitor) and chlorpromazine (the clathrin-mediated endocytosis inhibitor),^35^ affected the A1I2-1R2C18 or A1I2R2C18-mediated intracellular protein delivery processes. In the meantime, the cells pretreated with genistein inhibitor exhibited a slight reduction of cellular uptake, implying that the internalization mechanism is not mainly dependent on the caveolae-mediated endocytosis.^16^ This result was consistent with the energy-dependent endocytosis inhibitor sodium azide (NaN_3_),^21^ which had no apparent influence on cellular uptake behaviors. To further validate this view, the intracellular colocalization of BSA-FITC/A1I2R2C18 complexes was investigated. We observed that the intracellular signals of BSA-FITC showed slight overlap with the caveosomes or endo/lysosomes after HeLa cells were incubated with BSA-FITC/A1I2R2C18 complexes (Figure 3B,C), and their corresponding Pearson’s correlation coefficients were less than 0.4 (Caveolin-1) or 0.3 (Lysotracker) at the post-treatment of 0.5–8 h, respectively (Figure 3D). Besides, the effect of BSA-FITC/A1I2R2C18 complexes on the membrane integrity of endo/lysosomes was examined by the calcein and acridine orange staining assays,^36,37^ respectively. As indicated in Figure S14, compared to the BSA only, BSA/A1I2R2C18 complexes hardly showed any change in the membrane permeability of endo/lysosomes after treatment for 4 h. The results suggested the cellular uptake mechanism is entirely distinct from the classical endocytosis followed by endo/lysosome escape pathways.

It is well-known that lipid rafts are cholesterol-enriched heterogeneous and dynamic domains in which cholesterol has preferential interactions with the saturated lipids (such as sphingolipids) due to the hydrophobic force and hydrogen bonding.^38,39^ We observed that the cells pretreated with methyl-β-cyclodextrin (MβCD) dramatically inhibited most cellular uptake of complexes, which implied the uptake mechanism is primarily dependent on lipid raft-mediated translocation because of cholesterol depletion by MβCD.^40,41^ To further prove that the interaction between saturated alkyl tails of GAs and lipid raft domains could improve the cellular uptake efficiency, we synthesized oleic acid (C18-1) and linoleic acid (C18-2)-derived GAs with different degrees of saturation in the hydrophobic tails, namely, A1I2R2C18-1 and A1I2R2C18-2 (Figure 3E), respectively. We found that the corresponding BSA/A1I2R2C18-1 and BSA/A1I2R2C18-2 complexes showed very similar particle size, surface positive charge and BSA loading efficiency (>90%), as compared to the BSA/A1I2R2C18 (Figure S15). However, GAs with unsaturated alkyl tails significantly impeded cellular internalization, likely owing to the weak affinity of the unsaturated alkyl tails of GAs toward lipid raft domains (Figure 3F and Figure S16). This further demonstrated that the GA-mediated intracellular protein delivery was dependent on the lipid raft-associated translocation pathway. Besides, we performed the time-lapse imaging after HeLa cells were incubated with BSA-FITC/A1I2R2C18 complexes and recorded the images in a 30 s interval. We observed that a very quick intracellular protein delivery event, where the green fluorescence of BSA-FITC began to appear inside the cell at the initial 30 s and thoroughly spread all over the cytosol within another 30 s, as opposed to much longer cellular endocytosis and subsequent escape process (Figure 3G).^10,12,42^ This suggested that GA-mediated intracellular protein delivery is dominated by the passive membrane fusion mechanism. Our postulation was also confirmed by the fact that the preincubation under low temperature (4 °C), which would affect the membrane fluidity,^43^ greatly inhibited cellular uptake ability (Figure 3A). Overall, as depicted in Figure 3H, the entry mechanism is predominantly mediated by lipid raft-dependent membrane fusion for direct cytosolic delivery of biomacromolecules, bypassing the classical endocytic pathway.

### Cytosolic Delivery of Various Types of Proteins

**Figure 4:**
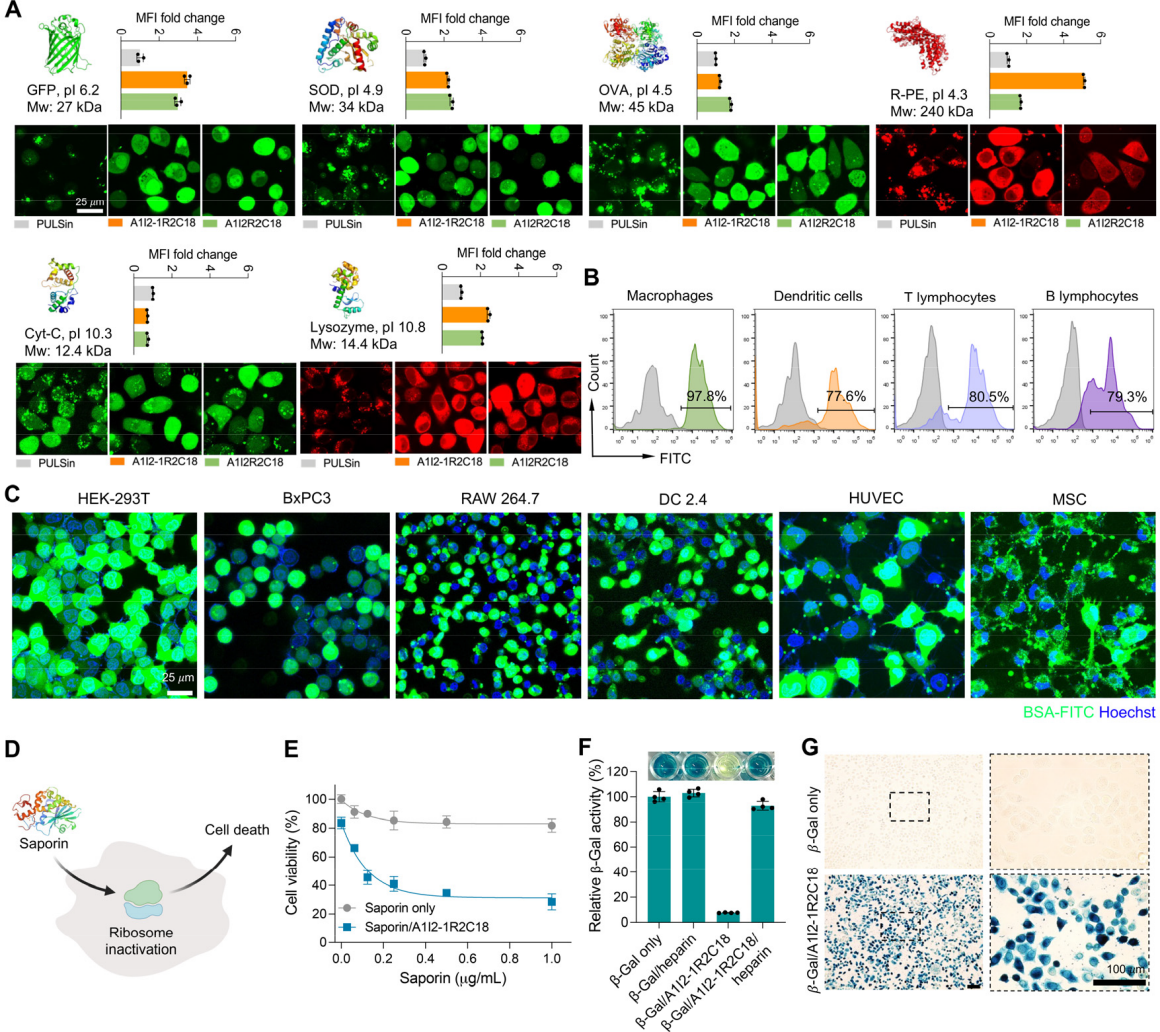
Intracellular delivery of various types of biomacromolecules. A) Intracellular delivery of negatively charged green fluorescence protein (GFP), superoxide dismutase (SOD), ovalbumin (OVA), R-phycoerythrin (R-PE), and positively charged cytochrome-C (Cyt-C) and lysozyme with the two GAs, A1I2-1R2C18, and A1I2R2C18, respectively. Proteins and GAs were at 8 μg/mL (*n* = 3). Scale bar is 25 μm. B) A1I2-1R2C18-mediated delivery of BSA-FITC into the primary macrophages, dendritic cells, T lymphocytes, and B lymphocytes. BSA-FITC and A1I2-1R2C18 were 8 μg/mL, respectively (*n* = 3). C) A1I2-1R2C18-mediated cytosolic delivery of BSA-FITC to various cell lines, including HEK-293T, BxPC3, RAW 264.7, DC 2.4, HUVEC and MSC cells. BSA-FITC and A1I2-1R2C18 were 8 μg/mL, respectively. Scale bar is 25 μm. D) The illustration of cytosolic saporin delivery leading to the inactivation of ribosomes and cell death. E) Cell viability of HeLa cells after incubation with various concentrations of saporin/A1I2-1R2C18 complexes. A1I2-1R2C18 was 6 μg/mL. (*n* = 4). F–G) In vitro β-Gal bioactivity of β-Gal/A1I2-1R2C18 complexes and the recovered β-Gal from β-Gal/A1I2-1R2C18 complexes in the presence of heparin (F), and (G) the intracellular β-Gal bioactivity as examined by the in situ X-Gal staining kit after HeLa cells incubated with β-Gal/A1I2-1R2C18 complexes for 4 h. β-Gal and A1I2-1R2C18 were 8 μg/mL, respectively. (*n* = 4). Scale bar is 100 μm.

To investigate the potential of GAs as the robust and versatile carriers for the intracellular delivery of various types of proteins, we first examined GA-mediated cytosolic delivery efficiency of protein cargos with different MWs and pIs, including negatively charged green fluorescence protein (GFP), superoxide dismutase (SOD-FITC), ovalbumin (OVA-FITC) and R-phycoerythrin (R-PE), and positively charged cytochrome-C (Cyt-C-FITC) and lysozyme (Lyso-RBITC). Generally, protein surfaces are chemically heterogeneous and contain cationic, anionic and hydrophobic amino acid residues; hence, they could coassemble with the GAs to form complexes through hydrophobic force and/or electrostatic interactions (Table S1). As shown in Figure 4A and Figures S17–S22, the two top-performing GAs, A1I2-1R2C18 and A1I2R2C18, could deliver various types of protein cargoes into the cytosol, exhibiting greater intracellular delivery efficiency than the PULSin (except for Cyt-C-FITC). We found that the MFI of R-PE treated by A1I2-1R2C18 was 3.1-fold higher than that treated with A1I2R2C18, suggesting GA with the shorter space linker I2-1 (A1I2-1R2C18) was more suitable to deliver high-molecular-weight proteins. In addition, we also demonstrated that A1I2-1R2C18 could successfully deliver BSA-FITC into various types of cell lines, including human embryonic kidney cells (HEK 293T), human pancreatic adenocarcinoma (BxPC3), murine macrophages (RAW 264.7), mouse dendritic cells (DC 2.4) and human umbilical vein endothelial cells (HUVEC), and primary mesenchymal stem cells (MSC) (Figure 4C). More importantly, A1I2-1R2C18 showed high efficacy to deliver BSA-FITC to the primary cells, including the macrophages, dendritic cells, T lymphocytes and B lymphocytes (Figure 4B, Scheme S1 and Figure S23), implying the promising perspective of GAs as delivery carriers for various therapeutic scenarios.

Next, we examined whether these delivered biomacromolecules remain bioactive after they are released in the cytoplasm. Saporin (pI: 9.3, MW: 32.8 kDa) is a cytotoxic protein that leads to cell death by denaturing cytosolic ribosomes (Figure 4D). As shown in Figure 4E, free saporin has generated minimal cell toxicity against HeLa cells because of its membrane-impermeable property. In contrast, saporin/A1I2-1R2C18 complexes showed dose-dependent cytotoxicity, which indicated that A1I2-1R2C18 could deliver saporin into the cytosol in the bioactive forms to lead to the inactivation of ribosomes. We also examined another model protein β-galactosidase (β-Gal; pI: 5.0, MW: 430 kDa), which is capable of catalyzing the substrate 5-bromo-4-chloro-3-indolyl-β-D-galactoside (X-Gal) into dark blue products. As shown in Figure 4F, we found that β-Gal temporally lost its bioactivity after complexation with A1I2-1R2C18. However, β-Gal recovered from β-Gal/A1I2-1R2C18 complexes still maintained high enzymatic function after the addition of heparin, which is a highly sulfated glycosaminoglycan with strong negative charges that can trigger the release of native β-Gal from the β-Gal/A1I2-1R2C18 complexes by competitive binding with the cationic A1I2-1R2C18. Furtherly, HeLa cells incubated with β-Gal/A1I2-1R2C18 complexes presented substantial dark blue products in the cytosol, which confirmed the robust bioactivity of intracellular β-Gal delivered by A1I2-1R2C18 (Figure 4G). Collectively, these confirmed that the GAs are a robust carrier platform for delivering a broad spectrum of proteins into the cytosol.

### Intracellular Delivery of Cas9 RNP for CRISPR-Cas9 Genome Editing in Vivo

**Figure 5:**
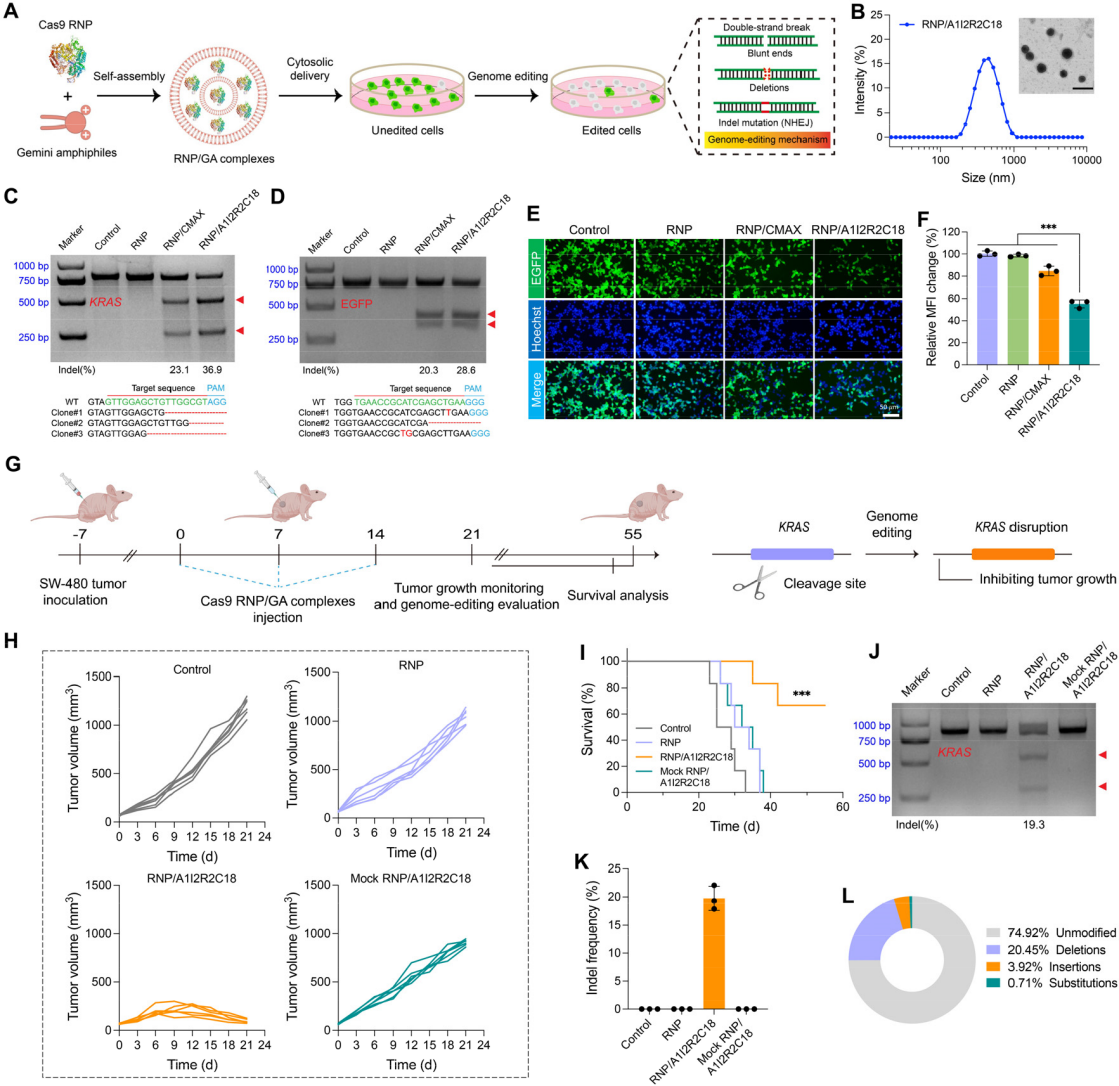
Cytosolic delivery of CRISPR/Cas9 ribonucleoprotein (RNP) for in vitro and in vivo genome editing. A) Schematic illustration of RNP/GA complexes mediated cytosolic delivery for targeting EGFP locus. B) Size distribution and TEM image of RNP/A1I2R2C18 complexes. Scale bar is 500 nm. C) T7E1 assay (upper panel) comes from intracellularly delivering RNP/A1I2R2C18 complexes in SW-480 cells (targeting *KRAS* locus). CMAX was designed as a positive control. Sanger sequence (lower panel) performed by T-A cloning at *KRAS* locus was obtained from SW-480 cells. Three sequences of clones with mutations were represented. Cas9 protein, sgRNA, and A1I2R2C18 were 2, 1, and 4 μg/mL, respectively. D) T7E1 assay (upper panel) comes from intracellularly delivering RNP/A1I2R2C18 complexes in 293T cells for targeting EGFP loci. Sanger sequence (lower panel) performed by T-A cloning at EGFP loci that obtained from 293T cells. Three sequences of clones with mutations were represented. Cas9 protein, sgRNA and A1I2R2C18 were 2, 1, and 4 μg/mL, respectively. E) Fluorescent images of 293T-EGFP cells treated with RNP/A1I2R2C18 complexes. Scale bar is 50 μm. F) Flow cytometry analysis of 293T-EGFP cells treated with RNP/A1I2R2C18 complexes via detecting the MFI. Cas9 protein, sgRNA, and A1I2R2C18 were 2, 1, and 4 μg/mL, respectively. ***P < 0.001. (*n* = 3). G) Schematic illustration of RNP/GA complexes mediated genome-editing therapy in SW480 tumor-bearing nude mice. H–I) Individual tumor growth (H) and survival rate (I) of nude mice in different treatment groups. (*n* = 6). ***P < 0.001 vs control. J–K) T7E1 assay of the mutant *KRAS* locus in the nude mice tumor. (*n* = 3). L) Deep sequencing results of *KRAS* mutation frequency after the RNP/A1I2R2C18 complexes mediated treatment.

The clustered, regularly interspaced, short palindromic repeats-associated Cas9 (CRISPR-Cas9) system has shown its potent ability to induce site-specific genome editing for the treatment of a wide range of genetic disorders. However, the development of efficient vectors for the intracellular delivery of Cas9 RNP remains a major challenge, due to its complicated complex structure composed of single-guide RNA and RNA-binding Cas9 endonuclease.^44,45^ In order to explore the ability of GAs to deliver Cas9 RNP intracellularly (Figure 5A), we conducted indel (insertions and deletions) mutation using T7 endonuclease I (T7E1) digestion assays to test the effectiveness of genome editing at the intended genome sites following Cas9 RNP delivery. First, we treated 293T cells with RNP/A1I2-1R2C18, RNP/A1I2R2C18 or RNP/A1I2-3R2C18 complexes, and found that A1I2R2C18 was the most efficient in delivering Cas9 RNP and leaded to an indel frequency of 37.9% in adeno-associated virus integration site 1 (*AAVS1*) loci (Figure S24). The result was further confirmed by the cellular uptake study in which A1I2R2C18-mediated intracellular delivery showed the highest fluorescence signal of the FITC-labeled Cas9 RNP (Figure S25). We found RNP/A1I2R2C18 complexes were in a narrow size distribution with a hydrodynamic size of ca. 459 nm (Figure 5B). Furthermore, the representative indel mutations by T7E1 assays and Sanger sequencing by T-A cloning analysis of different genomic locus, including *KRAS* in SW-480 cells (Figure 5C), *EGFP* in 293T-EGFP cells (Figure 5D), *AAVS1* and *HBB* (hemoglobin subunit beta) in 293T cells (Figure S26 and S27), collectively implied that A1I2R2C18 could mediate efficient genome editing at the target genomic locus, which is superior to commercially available delivery reagent CRISPRMAX Cas9 Transfection Reagent (CMAX). In the meantime, we observed that the green fluorescence signals of 293T-EGFP cells have been remarkably decreased upon the incubation of RNP/A1I2R2C18 targeting EGFP gene, leading to the decrease of 45% of GFP-positive cells (Figure 5E,F).

We further explored whether the disruption of mutant *KRAS* by in vivo delivery of RNP/A1I2R2C18 complexes could effectively treat nude mice bearing SW480 tumors (Figure 5G). As shown in Figure 5H and Figure S28, whereas the tumor-bearing mice treated with PBS, RNP, and mock RNP/A1I2R2C18 complexes exhibited rapid growth of tumor volume, the peritumoral administration of RNP/A1I2R2C18 complexes could effectively inhibit tumor growth, suggesting the efficient genomic disruption of mutant *KRAS*.^46^ There was no significant body weight change for the mice treated with RNP/A1I2R2C18 complexes, suggesting the safety profile of the treatment (Figure S29). In the meantime, we found that the treatment by RNP/A1I2R2C18 complexes significantly prolonged the mice survival rate (Figure 5I). As expected, significant indel mutation was detected in tumor tissues, and the indel frequency ranged from 17.9% to 22.1% (Figure 5J,K). The editing-induced mutation at *KRAS* genomic locus was further evidenced by Sanger sequencing (Figure S30), and the results showed significant deletions, insertions and substitutions at the targeted loci around the protospacer adjacent motif (PAM). Besides, the deep sequencing analysis of a single library generated from genomic DNA pooled from SW-480 solid tumors demonstrated significant mutations in the *KRAS* locus, consistent with the results from T7E1 assays (Figure 5L and Figure S31). The H&E-stained tumor tissue sections from the PBS, RNP, and mock RNP/A1I2R2C18 complexes treated groups showed hypercellular and obvious nuclear polymorphism (Figure S32A). In contrast, tumor-bearing mice treated with RNP/A1I2R2C18 complexes reduced tumor cells and enhanced tumor necrosis, as reflected by H&E staining. Similarly, the *K*i-67 staining assay showed that the treatment of RNP/A1I2R2C18 complexes significantly reduced tumor cell proliferation (Figure S32B). Collectively, our results demonstrated that the in vivo delivery of RNP/A1I2R2C18 complexes is effective for the treatment of primary SW-480 tumors, indicating their promising potential for therapeutic genome editing for cancer treatment.

## 3 Discussion

Multicomponent reactions provide a concise and powerful molecule discovery platform that can be introduced to combinatorially synthesize and screen carriers for intracellular protein delivery. We have shown that the library of GAs with similar skeletal structures, but different types of cationic heads, hydrophobic tails, and space linkers, can be quickly established by a Ugi four-component reaction. The synthesis of GAs by modular chemistry allows each component to be rationally designed and enables the generation of a carrier material library with high chemical diversity. Such a general strategy also helps us understand the structure–activity relationship and identify the key roles contributing to high delivery efficiency from 150 GA molecules. These findings provide a fast and efficient way of establishing a molecular library, which is useful for designing other types of small-molecule-based delivery carriers.

As opposed to the entry mechanism of conventional delivery carriers, GAs directly translocate proteins into cells mainly by means of lipid raft-dependent membrane fusion, bypassing the classical endocytic pathways. Despite their structural similarity to cationic lipids, the entry mechanism is entirely different from lipid nanoparticles or liposomes which mainly involve the endocytic pathways. Since particle sizes of protein/GA complexes are typically much larger than 200 nm, it is unlikely that they are mainly internalized by some endocytic pathways, such as clathrin-mediated endocytosis, fast endophilin-mediated endocytosis, and caveolin-mediated endocytosis.^47^ Furthermore, the rate of fluorescence spread is very fast upon the contact of FITC-BSA/GA complexes with cells (within 90 s), supporting the concept that GA-mediated delivery undergoes a membrane-fusion-type entry mechanism rather than endocytosis-dependent pathways. As lipid raft domains are transient and relatively ordered membrane regions that contain a large number of cholesterols; therefore, the substitution of unsaturated alkyl chain tails dramatically reduces the efficiency of GA-mediated cellular uptake due to the decrease of lipid raft-carrier interactions. The above evidence was further validated by the inhibition assay where the presence of MβCD inhibited the membrane fusion by depleting membrane cholesterol. As biomacromolecules are prone to be degraded by enzymes in the endo/lysosome compartments, the direct translocation of proteins into cells bypassing endocytic pathways would greatly promote delivery efficiency.

While small molecule-based delivery systems, e.g., lipid nanoparticles, usually require multicomponent formulations to load and stabilize the biomacromolecular cargoes, we have demonstrated that a single-component GA system is enough to mediate intracellular delivery in vitro and local delivery in vivo. By screening GA molecules with suitable ionizable headgroups, we note that a slightly positive charge under the physiological condition not only facilitates the interaction between GA carriers and biomacromolecular cargoes along with hydrophobic forces and/or electrostatic interactions, but also contributes to lower the cytotoxicity of carriers. Despite these promising results, it is unclear whether GAs are stable and efficient in vivo after systemic administration, and the issue of cell-type selectivity after systemic administration needs to be solved to reduce off-target toxicity; hence, their safety in vivo remains elusive despite their lower cytotoxicity in vitro. Therefore, future work will be dedicated to understanding the influence of surface functionalization on cell-type specific uptake and their safety, efficacy, and biodistribution in vivo, and exploring the possibilities to load other types of therapeutic biomacromolecules, such as antibodies, to broaden their application scopes.

## 4 Materials and Methods

### Materials

1,2-Bis(2-aminoethoxy)ethane, ethyl formate, isobutyraldehyde, 2-methylbutyraldehyde, 2-ethylbutyraldehyde, N,N-dimethylethylenediamine, N,N-dimethyl-1,3-propanediamine, N,N-diethyl-1,3-propanediamine, N-(3-aminopropyl)morpholine, 2-(2-aminoethyl)-1-methylpyrrolidine, lauric acid, tridecanoic acid, myristic acid, pentadecanoic acid, palmitic acid, heptadecanoic acid, stearic acid, nonadecanoic acid, arachidic acid, chlorpromazine hydrochloride and genistein were obtained from Tokyo Chemical Industry (TCI) Shanghai. 2,2′-Oxybis(ethylamine), diamino-3,6,9-trioxaundecane, phosphoryl chloride, calcein and triethylamine were purchased from Beijing InnoChem Science & Technology Co., Ltd. Chloroplatinic acid hexahydrate, methyl-β-cyclodextrin, amiloride hydrochloride and sodium borohydride were from Aladdin. β-Galactosidase was purchased from J&K Scientific Ltd. Bovine serum albumin (BSA), ovalbumin (OVA), lysozyme, saporin, cytochrome-C (Cyt-C), fluorescein isothiocyanate (FITC), rhodamine B isothiocyanate (RBITC), acridine orange hydrochloride hydrate and Hoechst 33342 were from Merk. Superoxide dismutase (SOD) was from Yili BioChem Company. R-Phycoerythrin (R-PE) was obtained from AAT Bioquest. Green fluorescent protein (GFP) was obtained according to the previously reported approach.^48^ CRISPR/Cas9 protein and CRISPR/Cas9-EGFP protein were obtained from the GenScript company. Dulbecco’s Modified Eagle’s Medium (DMEM), Opti-MEM and fetal bovine serum (FBS) were obtained from Gibco. Lysotracker red fluorescence probe was purchased from Yeasen Biotech Co., Ltd. In Situ β-galactosidase Staining Kit was from Beyotime Biotechnology. Recombinant Alexa Fluor 647 Anti-Caveolin-1 antibody was from Abcam. PULSin was obtained from Polyplus Transfection. Lipofectamine CRISPRMAX Cas9 (CMAX) transfection reagent and Pierce BCA Protein Assay Kit were from Thermo Fisher. T7 endonuclease I (T7E1) enzyme was obtained from New England Biolabs (Beijing) Ltd. sgRNAs were designed via using the online tool (http://chopchop.cbu.uib.no/). The transcription templates for in vitro transcription (IVT) of sgRNAs are given in Table S2. MEGAscript T7 Transcription Kit was from Invitrogen.

### Synthesis of 2,2′-Oxybis(ethylisocyanide)

2,2′-Oxybis(ethylamine) (1 g, 9.6 mmol) in ethyl formate (28.4 g, 384 mmol) was refluxed at 70 °C for 24 h. The solvent was removed in vacuo. The resulting formamide was dissolved in 15 mL DCM and then treated with triethylamine (9.7 g, 96 mmol). Then this solution was cooled in an ice bath, and phosphoryl chloride (4.4 g, 28.8 mmol) in 15 mL of DCM was added dropwise. After 3 h, the ice bath was removed, and K_2_CO_3_ was added and stirred for 1 h. The organic layer was separated, and after that, the aqueous layer was extracted with DCM. The combined organic layers were dried with K_2_CO_3_ and concentrated in vacuo. Purification by column chromatography (hexane and ethyl acetate, 2:3) and concentrated in vacuo to obtain pain yellow product (350 mg). ^1^H NMR (400 MHz, CDCl_3_): δ (ppm) = 3.58–3.68 (4 H, 2 CH_2_), 3.71–3.81 (4 H, 2 CH_2_). ^13^C NMR (400 MHz, CDCl_3_): δ (ppm) = 41.71, 68.66, 157.79 (NC).

### Synthesis of 1,2-Bis(2-isocyanoethoxy)ethane

1,2-Bis(2-aminoethoxy)ethane (10 g, 67.5 mmol) in ethyl formate (200 g, 2.7 mol) was refluxed at 70 °C for 24 h. The solvent was removed in vacuo. The resulting formamide was dissolved in 90 mL DCM and then treated with triethylamine (68.3 g, 675 mmol). Then this solution was cooled in an ice bath, and phosphoryl chloride (31 g, 202.5 mmol) in 90 mL DCM was added dropwise. After 3 h, the ice bath was removed, and K_2_CO_3_ was added and stirred for 1 h. The organic layer was separated, and after that, the aqueous layer was extracted with DCM. The combined organic layers were dried with K_2_CO_3_ and concentrated in vacuo. Purification by column chromatography (hexane and ethyl acetate, 2:3) and concentration in vacuo to obtain pain yellow product (3.5 g). ^1^H NMR (400 MHz, CDCl_3_): δ (ppm) = 3.53–3.63 (4 H, 2 CH_2_), 3.66–3.79 (8 H, 4 CH_2_). ^13^C NMR (400 MHz, CDCl_3_): δ (ppm) = 41.71, 68.66, 70.69, 157.41 (NC).

### Synthesis of 1,11-Diisocyano-3,6,9-trioxaundecane

Diamino-3,6,9-trioxaundecane (1 g, 5.2 mmol) in ethyl formate (15.4 g, 208 mmol) was refluxed at 70 °C for 24 h. The solvent was removed in vacuo. The resulting formamide was dissolved in 15 mL of DCM and then treated with triethylamine (5.3 g, 52 mmol). Then this solution was cooled in an ice bath, and phosphoryl chloride (2.4 g, 15.6 mmol) in 15 mL of DCM was added dropwise. After 3 h, the ice bath was removed, and K_2_CO_3_ was added and stirred for 1 h. The organic layer was separated, and after that, the aqueous layer was extracted with DCM. The combined organic layers were dried with K_2_CO_3_ and concentrated in vacuo. Purification by column chromatography (hexane and ethyl acetate, 1:4) and concentrated in vacuo to obtain pain yellow product (700 mg). ^1^H NMR (400 MHz, CDCl_3_): δ (ppm) = 3.56–3.62 (4 H, 2 CH_2_), 3.66–3.76 (12 H, 6 CH_2_). ^13^C NMR (400 MHz, CDCl_3_): δ (ppm) = 41.71, 68.66, 70.65, 70.86, 157.22 (NC).

### General Procedure for the Combinational Synthesis of GAs

Ugi four-component reactions for the synthesis of GAs were performed in a glass vial under gentle stirring, following the same general procedure, according to the previous reports with slight modifications.^27^ Briefly, 1 mmol of aldehydes and 1 mmol of amines were added to a glass vial containing 0.5 mL of methanol and reacted for 30 min at room temperature. Then, 1 mmol of carboxylic acids and 0.5 mmol of diisocyanides were orderly added, and the reaction mixture was reacted for 24 h at 40 °C. The resultant products were purified using column chromatography with methanol and dichloromethane as the eluent, then further characterized by the ESI-MS instrument (Bruker, times TOF) and ^1^H NMR spectroscopy (Bruker, AVANCE III 400 MHz). The critical aggregation concentration (CAC) of GAs was tested using the pyrene probe method.^49^

### Fluorescent Dye-Labeled Protein Synthesis

To obtain various fluorescent dye-labeled proteins, 10 mg/mL of FITC solution in DMSO was dropwise added into 10 mg/mL of various proteins solution in 10 mM phosphate-buffered saline buffer (PBS, pH 7.4), respectively, and the weight ratio of FITC to proteins was held at 1:10. Then, the reaction mixture was stirred for 24 h at room temperature under dark condition. To remove the unconjugated fluorescent dyes, the reacted solution was dialyzed in the dark against 10 mM PBS (pH 7.4) until no fluorescence in the dialysate, then further dialyzed against deionized water for another 24 h. The purified samples were lyophilized to produce FITC-labeled proteins, including FITC-labeled BSA (BSA-FITC), FITC-labeled SOD (SOD-FITC), FITC-labeled OVA (OVA-FITC) and FITC-labeled Cyt-C (Cyt-C-FITC). Similarly, RBITC-labeled lysozyme (Lyso-RBITC) was also obtained by the procedures described above. Subsequently, these fluorescent dye-labeled proteins were stored at −20 °C for further use.

### Protein/GA Complexes Formation and Characterization

For preparing the protein/GA complexes, GA was dissolved in ethanol at the concentration of 10 mg/mL, and then diluted with Hepes buffer (20 mM, pH 7.4) before use. The protein solutions including BSA, GFP, SOD, OVA, R-PE, Cyt-C, lysozyme, saporin, β-Gal, and Cas9 RNP were also prepared in Hepes buffers. Protein/GA complexes were obtained by simply mixing 30 μL of the GA solution with 20 μL of protein solution at various mass ratios and incubated for 1 min, followed by further dilution with 450 μL of serum-free DMEM. The particle size and ζ-potential of protein/GA complexes were performed on Zetasizer Nano ZS instrument (Malvern), and the morphology was observed by transmission electron microscope (TEM, JEOL-1400 Plus). To evaluate the protein binding efficiency, the protein/GA complexes were put into an ultrafiltration tube (100 kDa) and centrifugated at 3000 rpm for 30 min. The free protein content in the filtrate was measured by a Pierce BCA Protein Assay Kit.

### Cell Culture

HeLa (human cervical carcinoma), RAW 264.7 (murine macrophages), HEK 293T (human embryonic kidney cells), 293T-EGFP cells (established by infecting 293T cells with lentivirus harboring an EGFP expressing cassette) and MSC cells (mesenchymal stem cells) were cultured in DMEM mediums containing 10% FBS and 1% penicillin and streptomycin. DC 2.4 (mouse dendritic cells) and BxPC3 (human pancreatic adenocarcinoma) were cultured in 1640 mediums containing 10% FBS and 1% penicillin and streptomycin. SW-480 cells (human colon cancer) were cultured in Leibovitz’s L-15 medium containing 10% FBS and 1% penicillin and streptomycin. HUVEC (human umbilical vein endothelial cells) was cultured in endothelial cell media containing the supplement pack (PromoCell). All cells were incubated under 37 °C and 5% CO_2_ conditions.

### In Vitro Cytosolic Delivery of Protein Assay

Cells were seeded into 24-well plates at 1 × 10^5^ cells/well and incubated overnight. Then the culture medium was removed, and the wells were washed with PBS before the addition of protein/GA complexes. After incubation with protein/GA complexes for 4 h, the cells were collected, and tested by flow cytometry (Life Technology, Attune NxT). Similarly, the cells were incubated in glass-bottom dishes for the observation of intracellular fluorescence distribution via confocal laser scanning microscopy (Leica, SP8). According to the manufacturer’s introduction, the commercial protein delivery reagent PULSin was used as a positive control.

For transporting proteins into the primary immune cells, lymphocytes were isolated from mice spleen by Mouse Lymphocyte Separation Medium (Dakewe Biotech) according to the manufactory’s protocol. The lymphocytes were cultured in 24-well plates and incubated with BSA-FITC/GA complexes for 4 h. Then the macrophages, dendritic cells, T lymphocytes and B lymphocytes were dyed with anti-CD11b-AF700, anti-CD11c-PE, anti-CD3-PE-Cy7 and anti-CD19-APC, respectively. Then, the BSA-FITC positive cells were tested by flow cytometry.

### Cell Viability Study

HeLa cells were seeded into 96-well plates at 1 × 10^4^ cells/well and cultured overnight. The culture medium was removed, and the DMEM-diluted BSA-FITC/GA complexes were added into 96-well plates and incubated with cells for 4 h. After that, the sample solution was removed, and cells were incubated with fresh culture medium for another 20 h. Finally, the cell viability was tested by the standard MTT assay.

### Internalization Mechanism Study

HeLa cells were seeded into 24-well plates at 1 × 10^5^ cells/well and incubated overnight. In order to investigate the internalization mechanisms, the cells were pretreated with various endocytic inhibitors including 20 mM of sodium azide, 0.5 mM amiloride, 5 μg/mL chlorpromazine, 200 μg/mL genistein or 10 mM MβCD for 1 h, respectively. Then the inhibitors were removed, and the cells were incubated with protein/GA complexes for another 4 h at 37 °C. Besides, HeLa cells were also treated with protein/GA complexes at 4 °C for 4 h (Cells were pretreated at 4 °C for 15 min before incubation with complexes). Then, the cellular uptake was quantified by flow cytometry.

### Caveolin or Endosome Colocation Study

HeLa cells were seeded into glass-bottom dishes at 2 × 10^5^ cells/well and incubated overnight. After being washed with PBS, the cells were treated with BSA-FITC/A1I2R2C18 complexes for 0.5, 1, 2, 4, 6, or 8 h, respectively. For caveolin staining, HeLa cells were washed with PBS at each time point, then the cells were orderly fixed with 100% methanol (precooled under −20 °C) for 5 min, permeabilized with 0.1% Triton X-100 for 5 min and blocked with 1% BSA solution for 1 h, after that, HeLa cells were incubated with Alexa Fluor 647 Anti-Caveolin-1 antibody for 1 h at 37 °C. For endosome staining, HeLa cells were washed with PBS at predetermined time points and then incubated with Lysotracker Red for 30 min at 37 °C. Finally, the cells were counterstained with the Hoechst for 10 min and viewed under confocal laser scanning microscopy.

### Calcein Assay

HeLa cells were seeded into glass-bottom dishes and incubated overnight. After being washed with PBS, the cells were treated with DMEM containing 150 μg/mL of calcein with or without BSA/A1I2R2C18 complexes. After incubating for 4 h, the cells were washed with PBS to remove extracellular calcein, and then viewed under the CLSM.

### Acridine Orange Assay

HeLa cells were seeded into 24-well plates and incubated overnight. After being incubated with DMEM alone, BSA alone or BSA/A1I2R2C18 complexes for 4 h, the cell medium was removed, and cells were incubated with 2.5 μg/mL of acridine orange solution for another 15 min. The endosomal/lysosomal membrane permeability was tested by flow cytometry with the excitation at 488 nm, and the emission at 530 (green fluorescence) or 620 nm (red fluorescence), respectively.

### In Vitro Cytosolic Delivery of Toxic Saporin

HeLa cells were seeded into 96-well plates at 1 × 10^4^ cells/well and cultured overnight. The culture medium was removed, and the cells were washed with PBS and thereafter incubated with free saporin or saporin/A1I2-1R2C18 complexes at a series of saporin doses (0.0625, 0.125, 0.25, 0.5, 1 μg/mL) for 4 h, afterward, the test samples were removed, and cells were incubated with fresh culture medium for another 20 h. Finally, cell viability was tested by the MTT assay.

### Intracellular β-Gal Activity Assay

HeLa cells were seeded into glass-bottom dishes and incubated overnight. After being washed with PBS, the cells were incubated with β-Gal only, β-Gal/PULSin or β-Gal/A1I2-1R2C18 complexes for 4 h, and the intracellular β-Gal activity was examined via In Situ β-galactosidase Staining Kit, according to the manufacturer’s instruction. Then, the samples were observed under an optical microscope. For the in vitro β-Gal activity study, 125 μL of β-Gal solution or β-Gal/A1I2-1R2C18 complexes (8 μg/mL of β-Gal, 8 μg/mL of A1I2-1R2C18) were incubated with 62.5 μL X-gal contained working solution at 37 °C for 1 h in a 96-well plate, the resulting product was dissolved in DMSO and the absorbance at 633 nm was measured by a microplate reader (SYNERGY, BioTek). Besides, in order to investigate the enzyme bioactivity of the released β-Gal, 10 μL β-Gal/A1I2-1R2C18 complexes were diluted with 115 μL heparin sodium (0.1 mg/mL) and incubated for 1 h before being treated with X-gal.

### Delivery of Cas9 RNP for In Vitro CRISPR/Cas9 Genome Editing

293T cells and SW-480 cells were seeded into 24-well plates at 1 × 10^5^ cells/well and cultured overnight. The RNP/GA complexes were prepared by mixing CRISPR/Cas9 ribonucleoprotein (RNP) with GAs for 1 min at room temperature and then diluted with serum-free DMEM or Leibovitz’s L-15 medium, the concentration of Cas9 protein, sgRNA and GA were 2, 1 and 4 μg/mL. After that, the cell culture medium was removed, the cells were washed with PBS, and then the RNP/GA complexes solution was added to the wells. After incubating for 4 h, the tested samples were replaced with fresh culture mediums and incubated for another 48 h before analysis. The CRISPRMAX Cas9 (CMAX) transfection reagent was chosen as a positive control and utilized by the manufacturer’s protocol.

### T7 Endonuclease I (T7E1) Assay

According to the experimental procedures described above, the T7E1 assay was performed to measure the disruption in the *AAVS1*, *HBB*, EGFP and *KRAS* genome loci. Simply put, the genomic DNA was obtained by harvesting transfected cells and tissues using the Trelief Animal Genomic DNA Kit (TSINGKE Biological Technology). Then, the genomic regions flanking the target site of Cas9 were amplified by PCR (Table S3), and then the TIANquick Midi Quantification Kit (TIANGEN BIOTECH) was used for DNA purification. 200 ng of purified PCR products was utilized to carry out the T7E1 assay, then these products were analyzed by agarose gel electrophoresis, followed by imaging by a gel documentation system. ImageJ was used to calculate undigested and digested bands of gray levels. The indel percentage was calculated using the following formula: [1 – (1–fraction cleaved)^1/2^] × 100%, in which the fraction cleaved represents the intensity of the cleaved band compared to the intensity of both the cleaved and uncleaved bands, after digestion with T7 endonuclease I. To perform T-A cloning and Sanger sequencing, we followed the protocol provided by TSINGKE Co., Ltd. Specifically, the amplified DNA fragment was cloned into the T vector and subsequently transformed into DH5α. Monoclonal was selected and sent for Sanger sequencing at Youkang Biotech Co., Ltd. The obtained DNA sequences were aligned with the target-gene locus using SnapGene software for analysis.

### In Vivo Treatment Efficacy Evaluation

All the animals were from Shanghai SLAC Laboratory Animal Co., Ltd. (Shanghai, China) and were fed in the Laboratory in Animals Centre, Zhejiang University. (Hangzhou, China). The animal experiments were performed according to the NIH guidelines for the care and use of experimental animals and were approved by the Laboratory Animal Welfare and Ethics Committee of Zhejiang University. In the SW-480 xenografts primary tumor model, SW-480 cells (1 × 10^6^) were injected subcutaneously into the right flank of BALB/c nude mice (day −7). On day 0, when tumors reached a size of about 50–80 mm^3^, the mice were randomly divided into 4 groups (6 mice per group) and treated with PBS, RNP, RNP/A1I2R2C18 or mock RNP/A1I2R2C18 complexes, respectively. The doses of Cas9 protein, sgRNA, and A1I2R2C18 in each mouse were 1, 0.5, and 2 mg/kg, respectively. The complexes were injected into the mice through peritumor injection on days 0, 7, and 14. The tumor volume and body weights of mice were measured during the treatment. Tumor volume was calculated by the formula: tumor volume = 0.5 × length × width^2^. The tumor-bearing mice were sacrificed on day 21, and the tumor tissues were collected and fixed with 4% paraformaldehyde for 48 h at 4 °C. In the survival study, mice were sacrificed when the volume of tumor higher than 1.5 cm^3^ or when the mice was moribund. The survival rate was analyzed from day 0 to day 55. Animal care technicians were blinded to the treatment groups. In the histological assay, tumor sections were stained with hematoxylin and eosin (H&E). *Ki*-67 immunohistochemical staining was applied to evaluate tumor cell proliferation according to the manufacturer’s instructions.

### Deep Sequencing Assay

A deep sequencing assay was conducted using the following procedure. Evaluation and design of off-target loci were performed using the CasOFFinder Web site (www.rgenome.net/cas-offinder/). Amplification of corresponding fragments was accomplished by utilizing specific primers (Table S4), referred to as the first PCR product. Following the amplification of corresponding fragments designed by the CasOFFinder Web site, PCR/Gel Extraction and Purification kits (Vazyme Biotech Co., Ltd.) were utilized to purify the resulting product. The purified product was subsequently subjected to further amplification to generate PCR fragments with a size limit of 250 base pairs (bp), encompassing the target gene loci, and referred to as the second PCR product. The extracted and purified product from the previous step underwent further amplification using primers containing an index sequence. The resulting product was again purified using PCR/Gel Extraction and Purification kits. Finally, the products underwent sequencing analysis, and the obtained data were analyzed using CRISPResso2 software based on the provided instructions.

### Statistics and Reproducibility

All experimental data were presented as mean ± standard deviation (SD) and performed at least three times. Group analysis was carried out using one-way ANOVA by GraphPad Prism (8.0). *P* < 0.05 (*) was considered as a significant difference.

## Supporting Information Available

In vitro intracellular protein delivery screening, impact trend of the structure of GAs; particle size, ζ potential, protein loading efficiency, TEM images, and CLSM images; GA-dose dependent cell cytotoxicity and IC50 values; ^1^H NMR spectrum and mass spectrum; CAC values; calcein assay and acridine orange assay; scheme and FACS analysis of primary cells from spleen; T7E1 assay, tumor growth, body weight changes, sanger sequence, deep sequencing analysis, H&E-stained section, and *Ki*-67 immunohistochemical section; sequences of sgRNA, sequences for PCR amplification and sequences for deep sequencing analysis (PDF)
Transparent Peer Review report available (PDF)
