# Supplementary material for: Direct Cytosolic Delivery of Proteins and CRISPR-Cas9 Genome Editing by Gemini Amphiphiles via Non-Endocytic Translocation Pathways
Source: ACS Cent Sci. 2023 Jun 8;9(7):1313–26. doi: 10.1021/acscentsci.3c00207 (PMC10375873; doi:10.1021/acscentsci.3c00207)
Supplement: Supplementary file 1 — oc3c00207_si_001.pdf [file oc3c00207_si_001.pdf]

Supporting Information

Direct Cytosolic Delivery of Proteins and CRISPR-Cas9 Genome Editing by Gemini Amphiphiles via Non-Endocytic Translocation Pathways

Zhicheng Le[1,†], Qi Pan[2,†], Zepeng He[1], Hong Liu[1], Yi Shi[1], Lixin Liu[1], Zhijia Liu[1,*], Yuan Ping[2,*] and Yongming Chen[1,*]

[1]School of Materials Science and Engineering, Key Laboratory for Polymeric Composite and Functional Materials of Ministry of Education, Sun Yat-sen University, Guangzhou 510006, China.

[2]College of Pharmaceutical Sciences, Zhejiang University, Hangzhou 310058, China.

[†]These authors contributed equally to this work.

*Corresponding authors: E-mail: chenym35@mail.sysu.edu.cn; pingy@zju.edu.cn; liuzhj9@mail.sysu.edu.cn

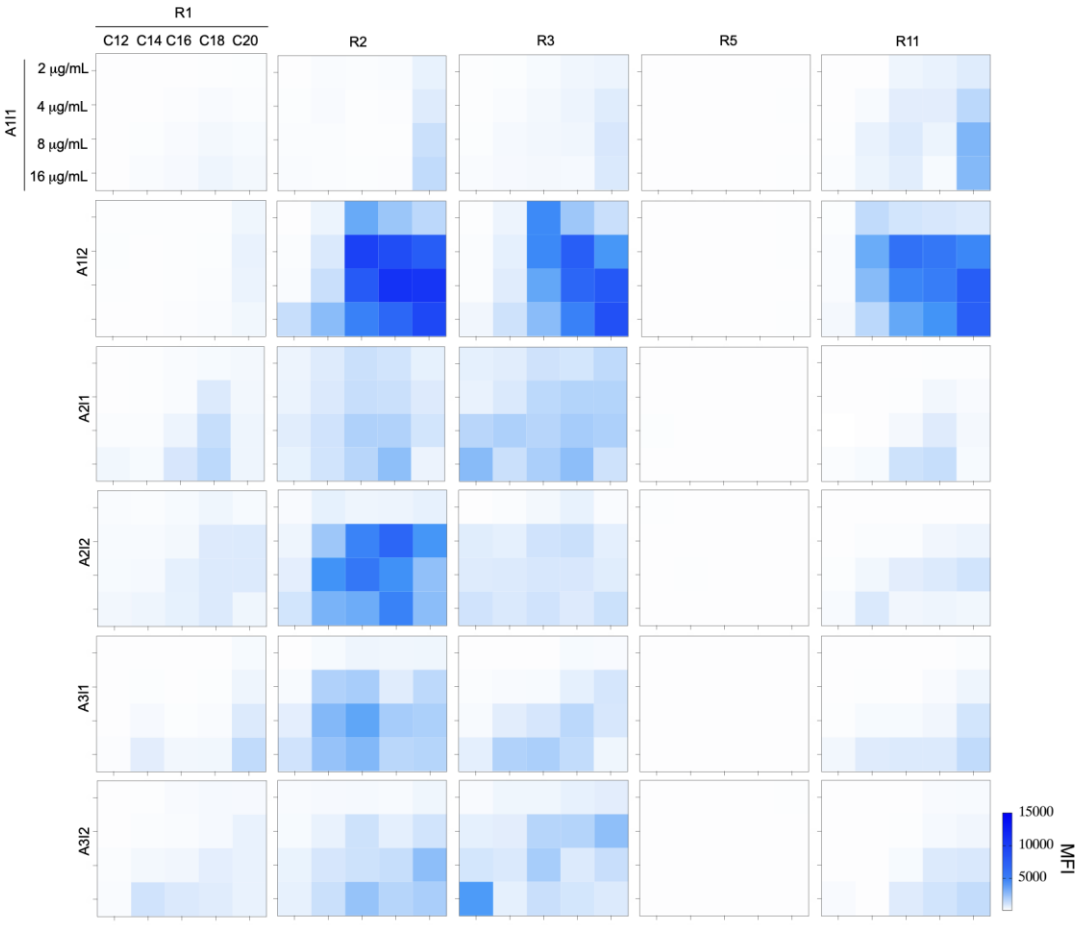

Figure S1. Intracellular mean fluorescence intensity (MFI) after HeLa cells incubated with various BSA-FITC/GA complexes for 4 h. BSA-FITC was kept at 4 μg/mL, and each GA was 2, 4, 8 or 16 μg/mL, respectively (n = 3).

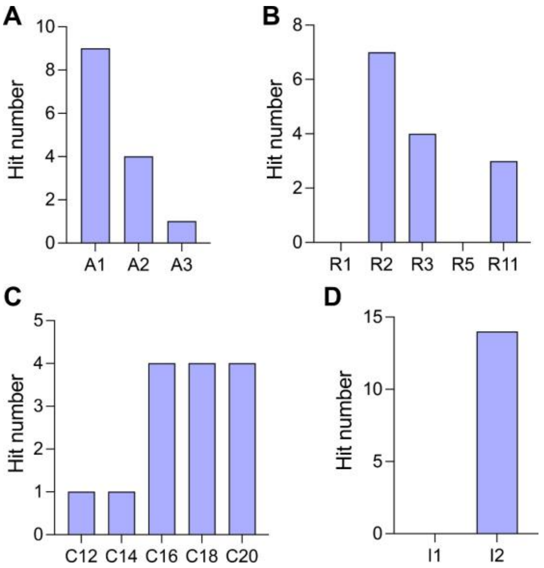

Figure S2. The impact of aldehydes (A), amines (B), carboxylic acids (C) and diisocyanides (D) on the hit number of GAs. The intracellular MFI from HeLa cells treated by GAs higher than the PULSin was counted as the hit GA.

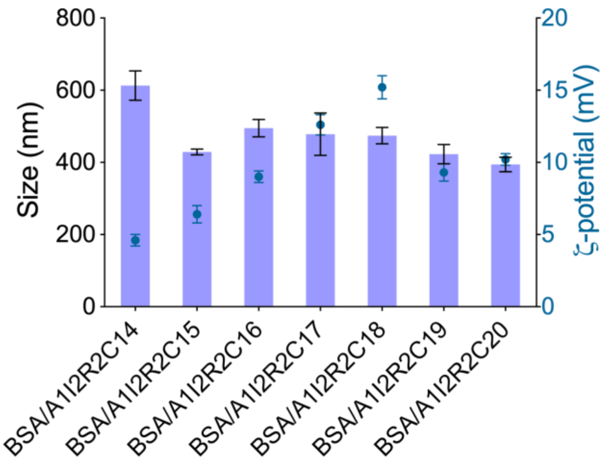

Figure S3. Particle diameter and ζ-potential values of various BSA/GA complexes. BSA was 4 μg/mL and GAs were 8 μg/mL (n = 3).

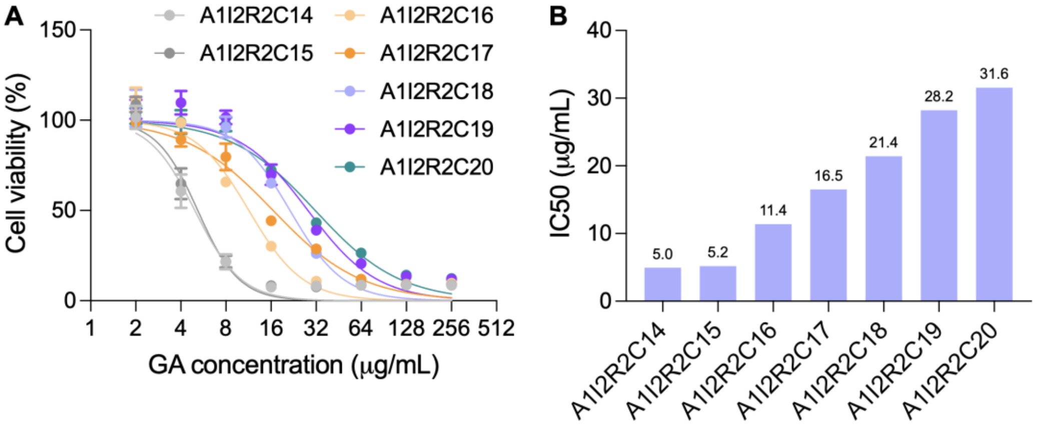

Figure S4. GA-dose dependent relative cell viability (A) and the corresponding 50% inhibitory concentration (IC50) values of each GA (B) (n = 4).

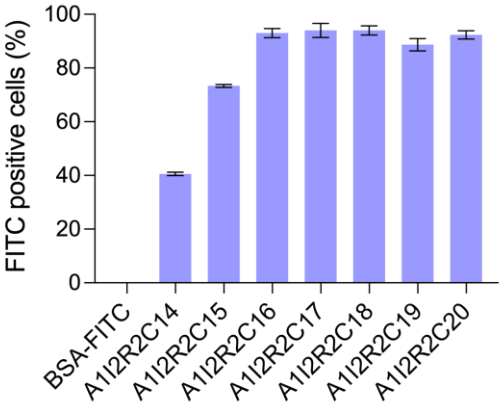

Figure S5. BSA-FITC positive cells percentage after HeLa cells treated with various BSA-FITC/GA complexes. BSA-FITC only treated cells were set as a negative control. BSA-FITC was 4 μg/mL, and GAs (A1I2R2C14–A1I2R2C20) were 8 μg/mL (n = 3).

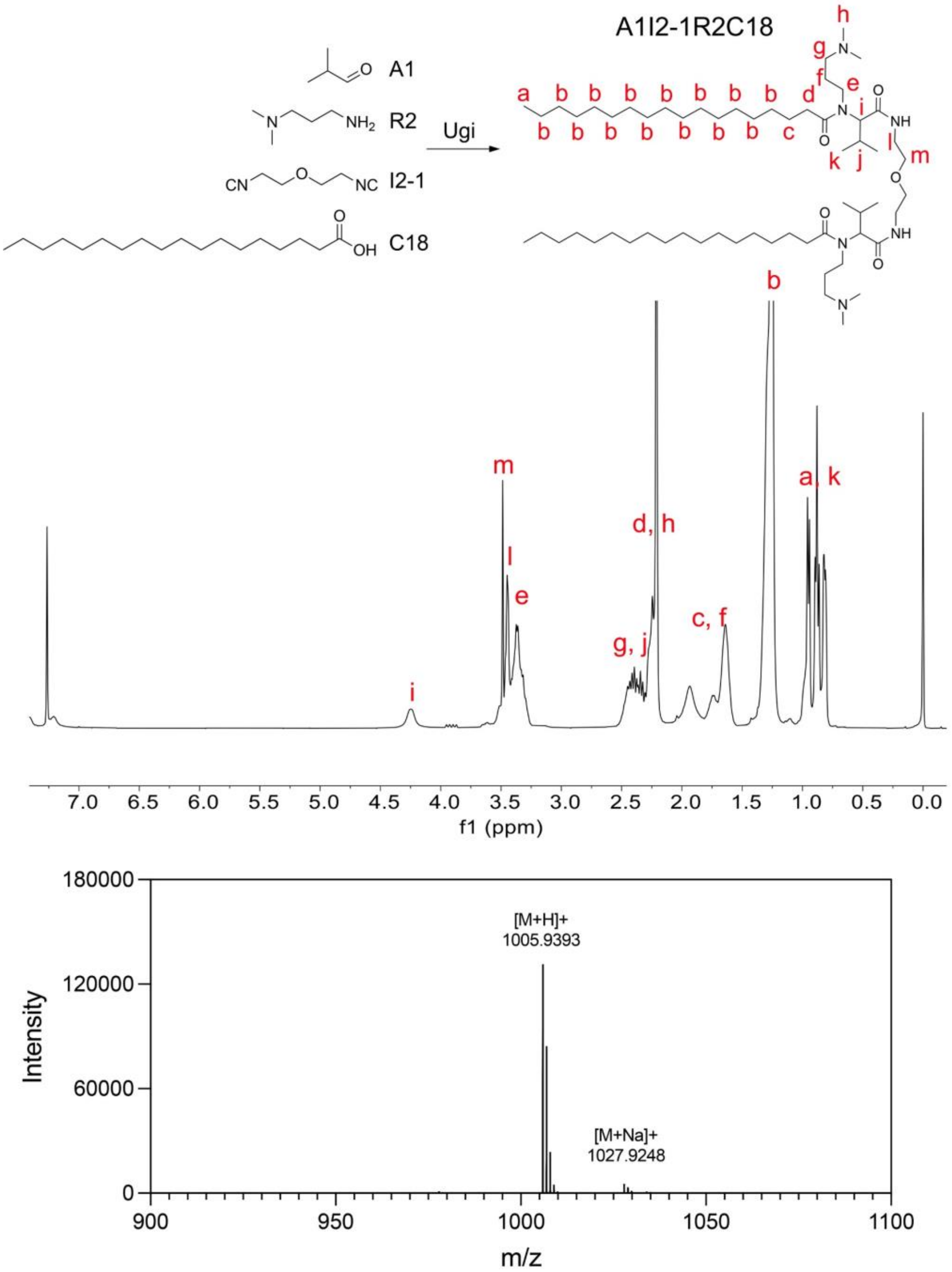

Figure S6. ^{1}H-NMR spectrum and mass spectrum of A1I2-1R2C18. The solvent is $CDCl_3$ for NMR measurement.

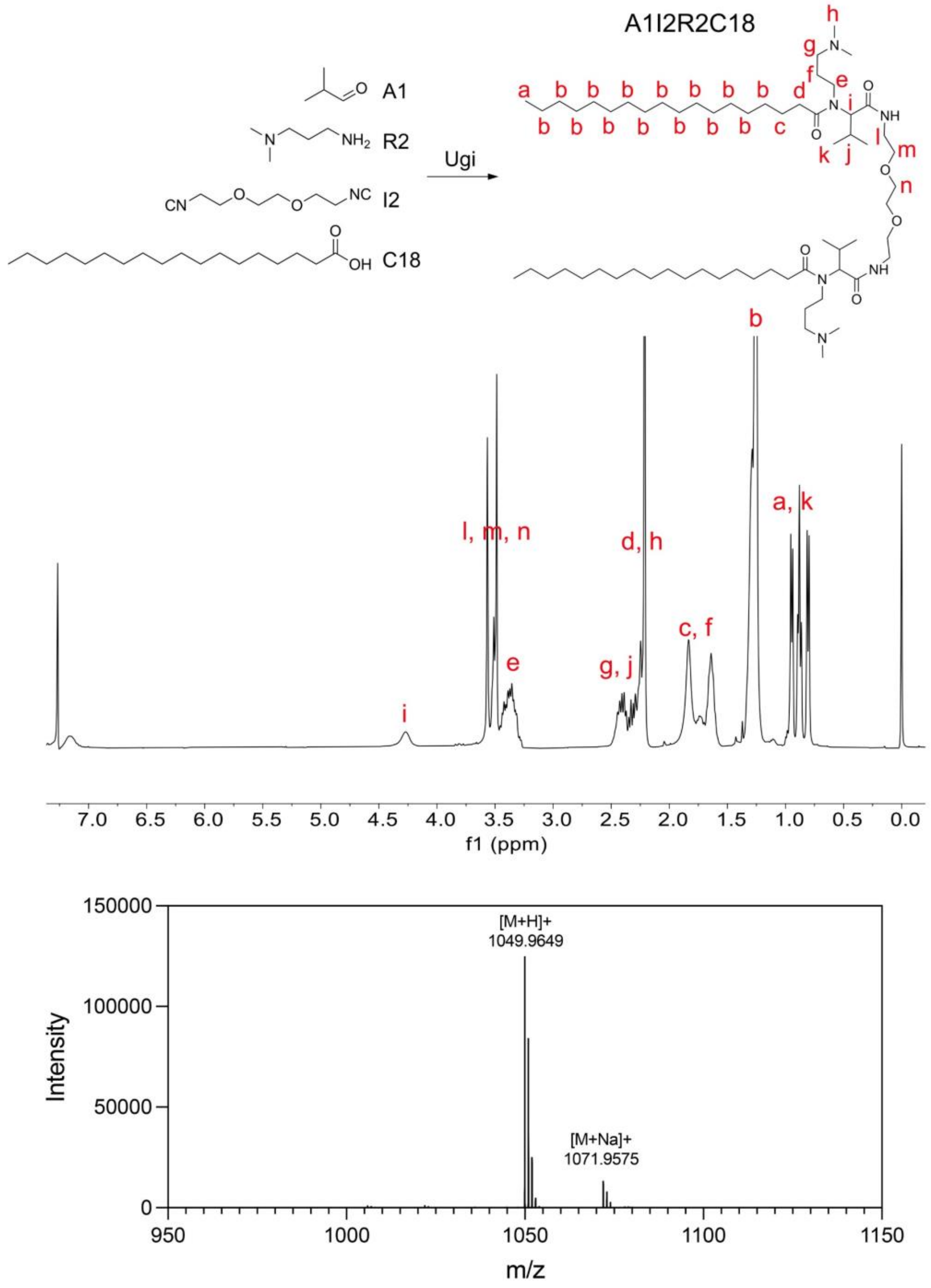

Figure S7. ^{1}H-NMR spectrum and mass spectrum of A1I2R2C18. The solvent is $CDCl_3$ for NMR measurement.

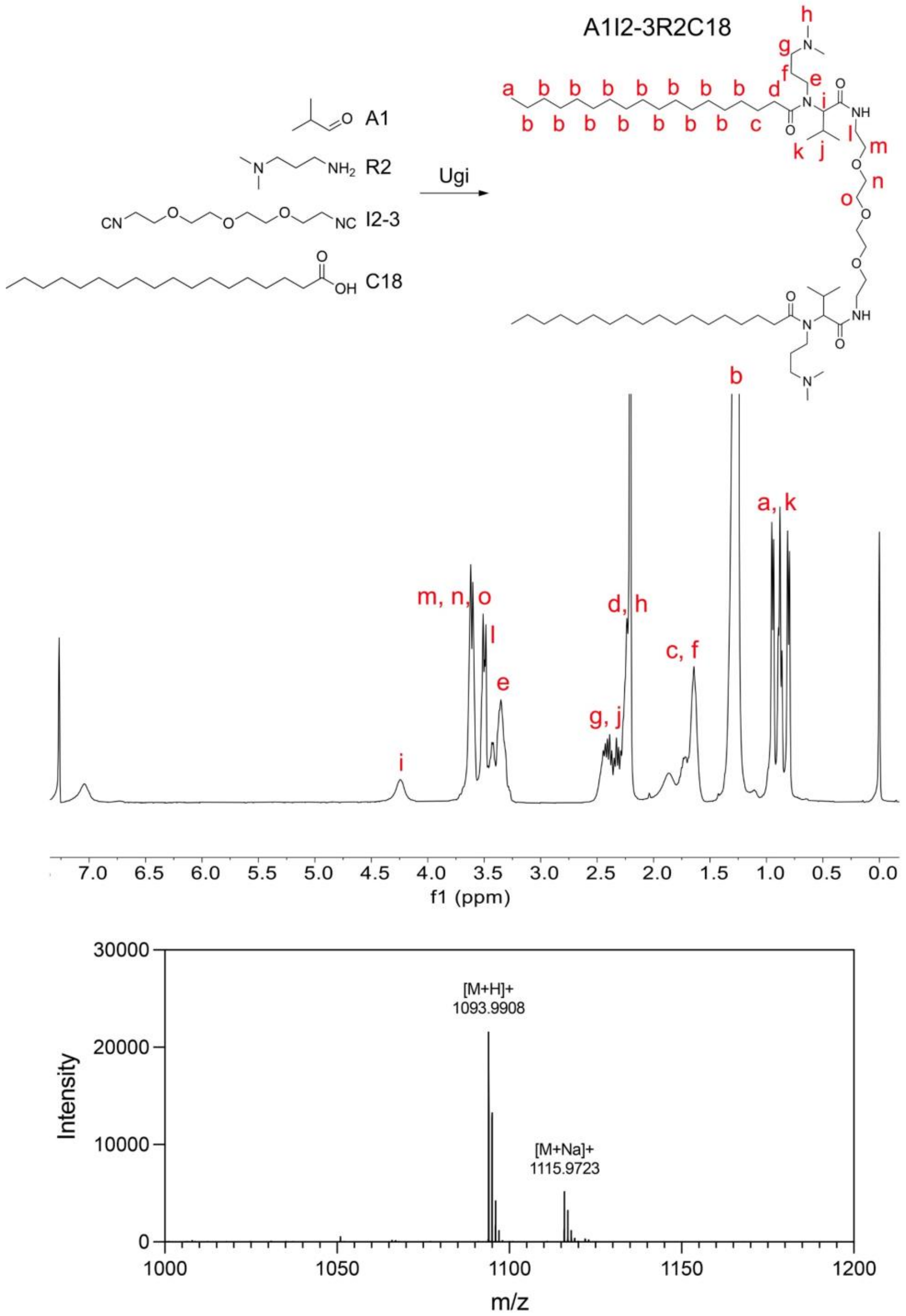

Figure S8. ^{1}H-NMR spectrum and mass spectrum of A1I2-3R2C18. The solvent is $CDCl_3$ for NMR measurement.

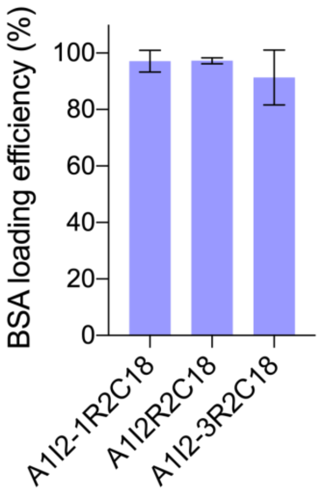

Figure S9. The protein loading efficiency of BSA/A1I2-1R2C18, BSA/A1I2R2C18 and BSA/A1I2-3R2C18 complexes (n = 4).

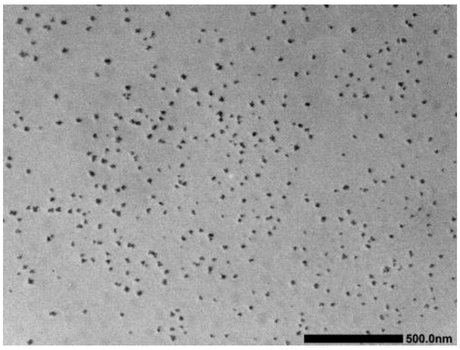

Figure S10. TEM image of BSA-Pt, scale bar is 500 nm.

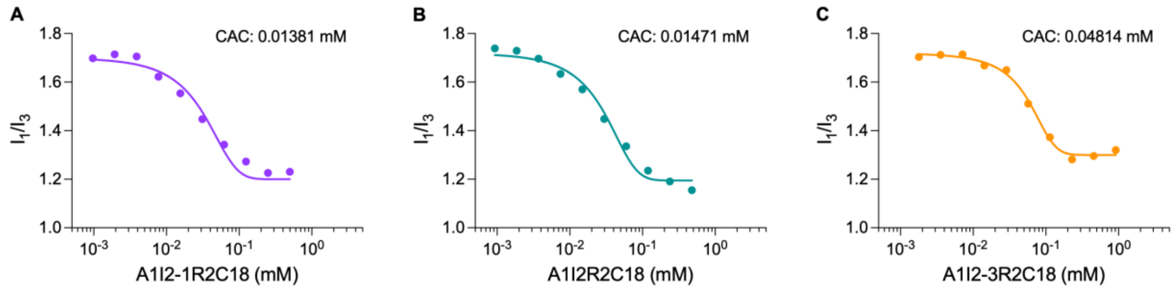

Figure S11. Critical aggregation concentration (CAC) of A1I2-1R2C18 (A), A1I2R2C18 (B), and A1I2-3R2C18 (C) as determined by the pyrene probe method.

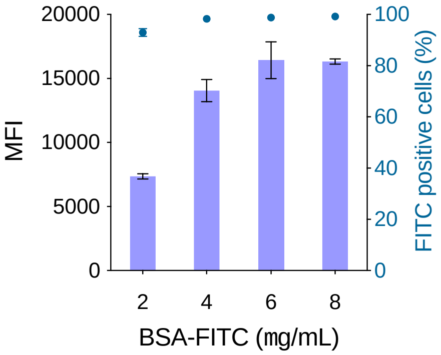

Figure S12. MFI and BSA-FITC positive cells after HeLa cells incubated with BSA-FITC/A1I2R2C18 complexes at varied BSA-FITC concentrations for 4 h. A1I2R2C18 was kept constant at 8 μg/mL (n = 3).

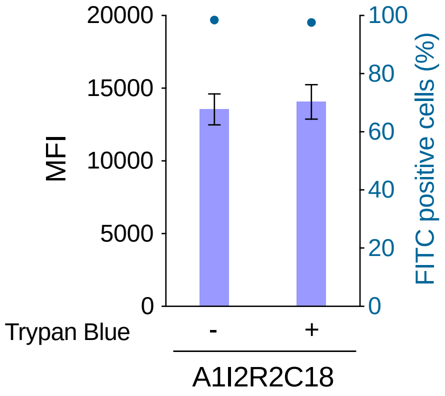

Figure S13. MFI and BSA-FITC positive cells of BSA-FITC/A1I2R2C18-transfected HeLa cells before and after trypan blue quenching. Trypan blue was 0.4 mg/mL, BSA-FITC was 4 μg/mL and A1I2R2C18 was 8 μg/mL (n = 3).

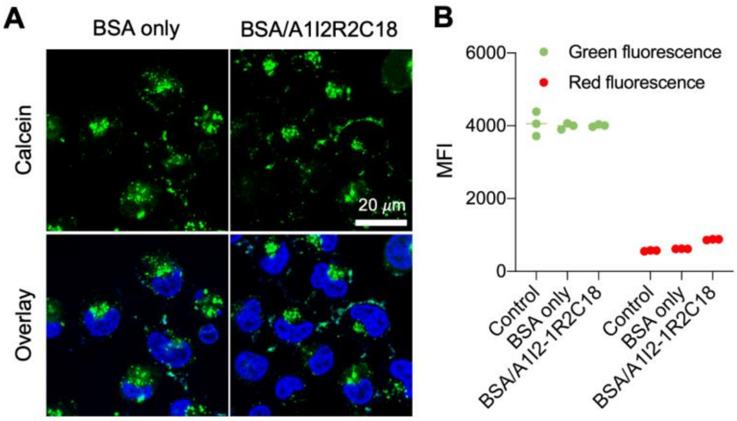

Figure S14. HeLa cells treated with calcein (A) or acridine orange (B) for examining the membrane permeability of endo/lysosomes. BSA-FITC was 4 μg/mL and A1I2R2C18 was 8 μg/mL (n = 3). Scale bar is 20 μm.

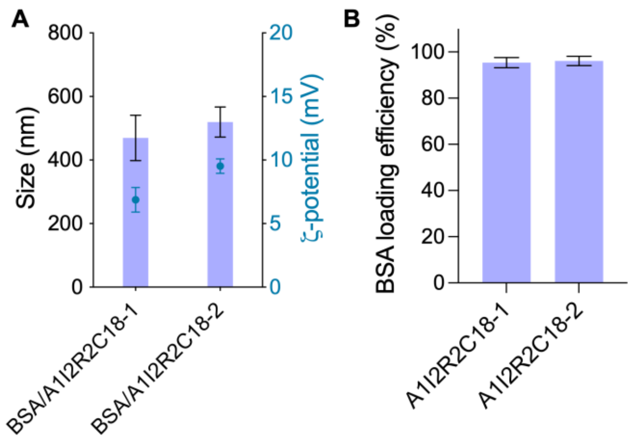

Figure S15. A) Particle sizes and ζ-potentials of BSA/A1I2R2C18-1 and BSA/A1I2R2C18-2 complexes (n = 3). B) Protein loading efficiency of BSA/A1I2R2C18-1 and BSA/A1I2R2C18-2 complexes (n = 4). BSA was 4 μg/mL, and A1I2R2C18-1 and A1I2R2C18-2 were 8 μg/mL, respectively.

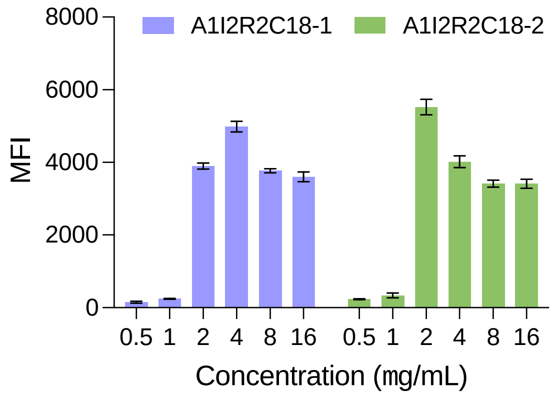

Figure S16. MFI of HeLa cells treated with BSA-FITC/A1I2R2C18-1 or BSA-FITC/A1I2R2C18-2 complexes for 4 h. BSA-FITC was 4 μg/mL, A1I2R2C18-1 and A1I2R2C18-2 were 0.5, 1, 2, 4, 8 or 16 μg/mL, respectively (n = 3).

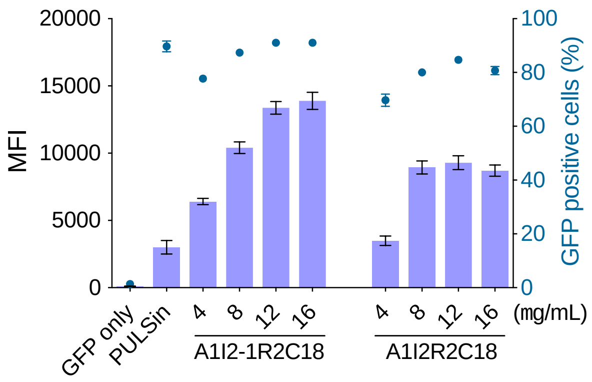

Figure S17. MFI and GFP positive cells after HeLa cells incubated with GFP/A1I2-1R2C18 or GFP/A1I2R2C18 complexes for 4 h. The GFP was 8 μg/mL (n = 3).

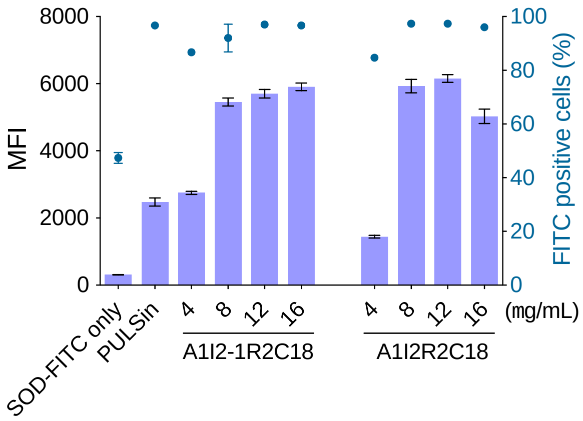

Figure S18. MFI and SOD-FITC positive cells after HeLa cells incubated with SOD-FITC/A1I2-1R2C18 or SOD-FITC/A1I2R2C18 complexes for 4 h. The SOD-FITC was 8 µg/mL (n = 3).

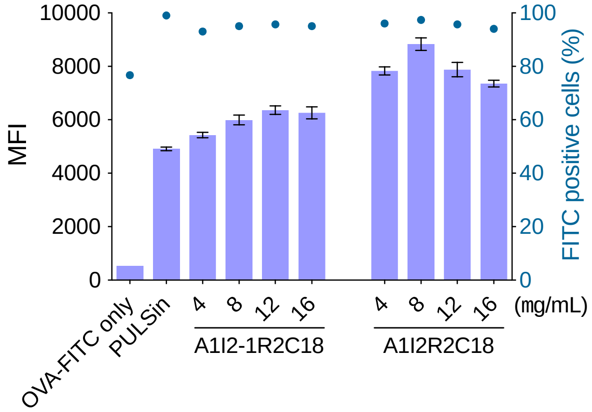

Figure S19. MFI and OVA-FITC positive cells after HeLa cells incubated with OVA-FITC/A1I2-1R2C18 or OVA-FITC/A1I2R2C18 complexes for 4 h. The OVA-FITC was 8 µg/mL (n = 3).

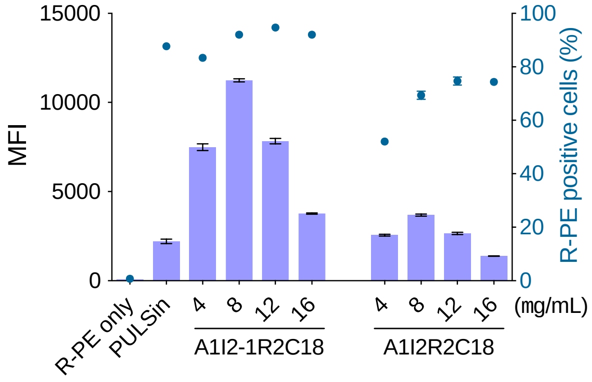

Figure S20. MFI and R-PE positive cells after HeLa cells incubated with R-PE/A1I2-1R2C18 or R-PE/A1I2R2C18 complexes for 4 h. The R-PE was 8 µg/mL (n = 3).

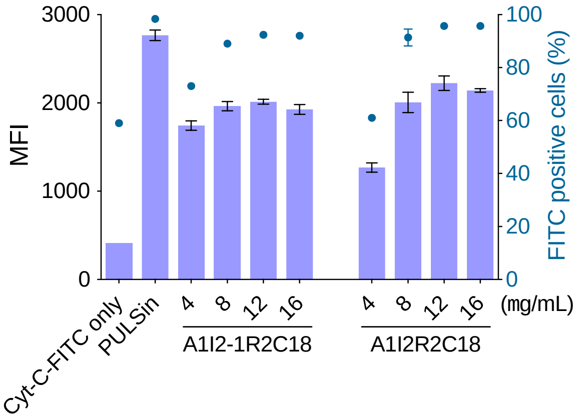

Figure S21. MFI and Cyt-C-FITC positive cells after HeLa cells incubated with Cyt-C-FITC/A1I2-1R2C18 or Cyt-C-FITC/A1I2R2C18 complexes for 4 h. The Cyt-C-FITC was 8 μg/mL (n = 3).

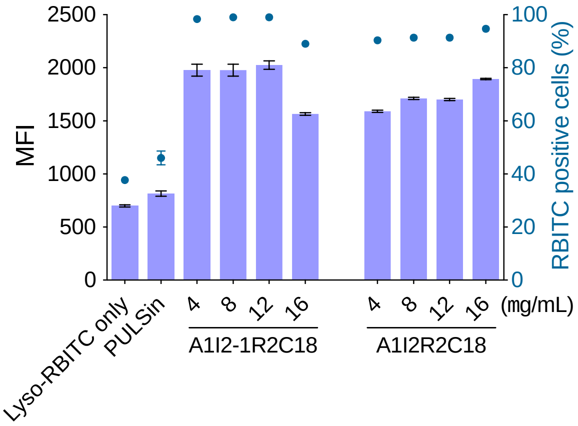

Figure S22. MFI and Lyso-RBITC positive cells after HeLa cells incubated with Lyso-RBITC/A1I2-1R2C18 or Lyso-RBITC/A1I2R2C18 complexes for 4 h. The Lyso-RBITC was 8 μg/mL (n = 3).

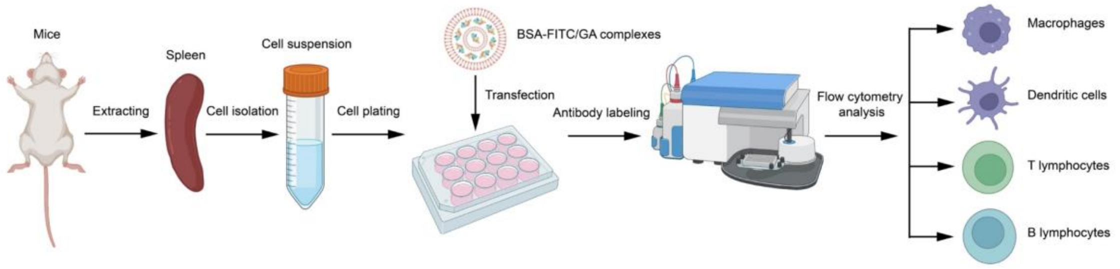

Scheme S1. In vivo isolation of macrophages, dendritic cells, T lymphocytes and B lymphocytes, and intracellular protein delivery to the primary immune cells.

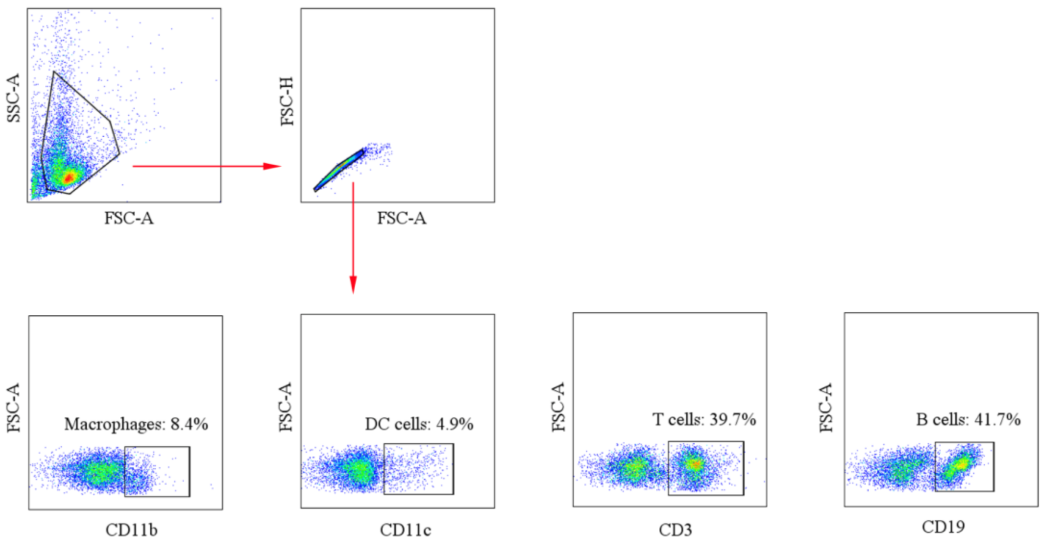

Figure S23. Representative FACS analysis to determine CD11b, CD11c, CD3 and CD19 positive cells from mice spleen.

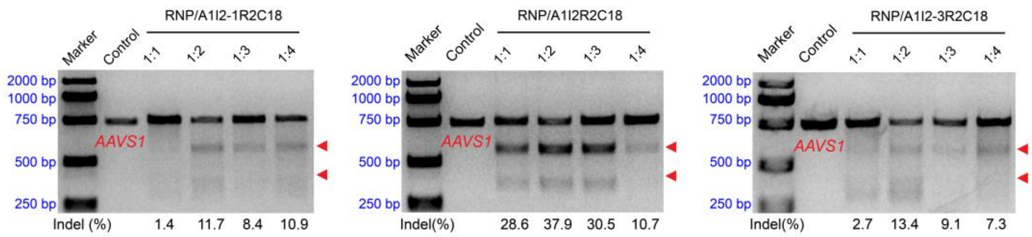

Figure S24. T7E1 assay results from the intracellular delivery of Cas9 RNP/GA complexes targeting *AAVS1* gene in 293T cells. Cas9 protein was 2 µg/mL, sgRNA was 1 µg/mL, GA was 2, 4, 6 or 8 µg/mL, respectively. Cas9 ribonucleoproteins (RNP) concentration was defined as 2 µg/mL according to the used Cas9 protein.

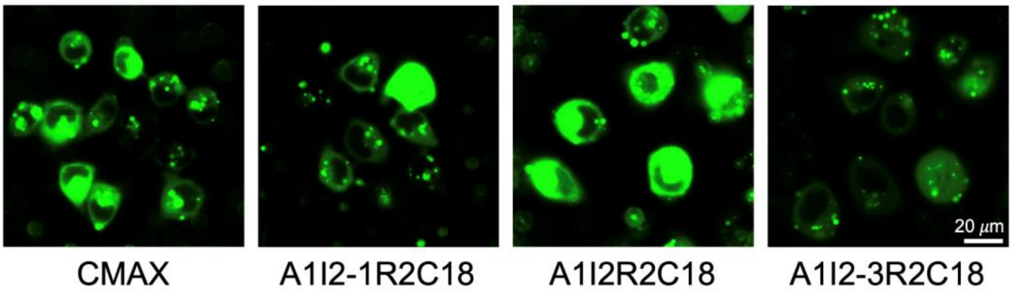

Figure S25. Representative images of 293T cells incubated with Cas9 RNP/CMAX or Cas9 RNP/GA complexes for 4 h, Cas9 protein was labelled with FITC. Cas9 protein was 2 µg/mL, sgRNA was 1 µg/mL, and GA was 4 µg/mL. Scale bar is 20 µm.

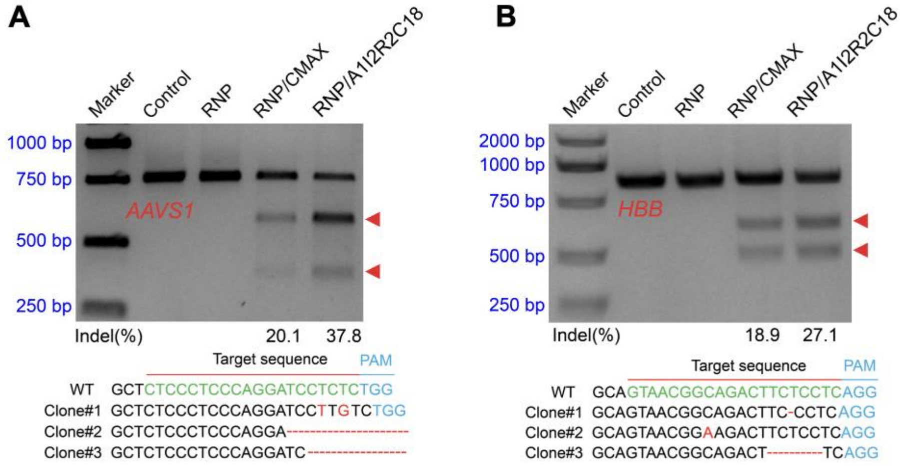

Figure S26. T7E1 assay (upper panel) comes from intracellularly delivering RNP/A1I2R2C18 complexes in 293T cells for targeting *AAVS1* (A) and *HBB* loci (B). CMAX was designed as positive control. Sanger sequence (lower panel) performed by T-A cloning at *AAVS1* and *HBB* loci that were obtained from 293T cells. Three sequences of clones with mutations were represented. Cas9 protein was 2 µg/mL, sgRNA was 1 µg/mL, and A1I2R2C18 was 4 µg/mL.

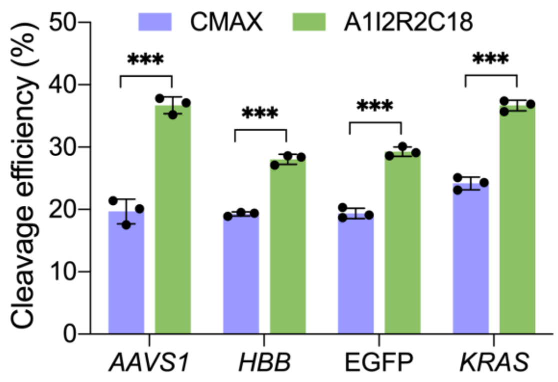

Figure S27. Quantitative cleavage efficiency of four gene locus (*AAVS1*, *HBB*, EGFP and *KRAS* locus) by means of T7E1 assay and ImageJ. ***$P < 0.001$ (n = 3). Cas9 protein was 2 µg/mL, sgRNA was 1 µg/mL, and A1I2R2C18 was 4 µg/mL.

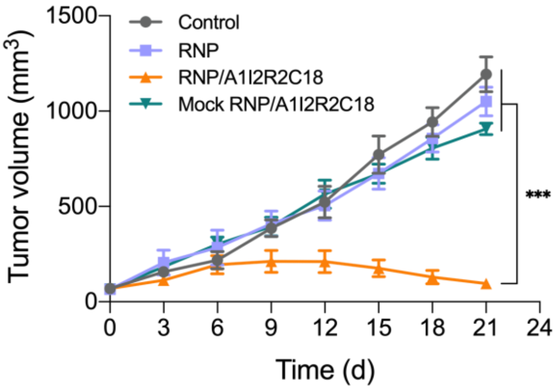

Figure S28. Tumor growth of nude mice in different treatment groups. ***$P < 0.001$ (n = 6).

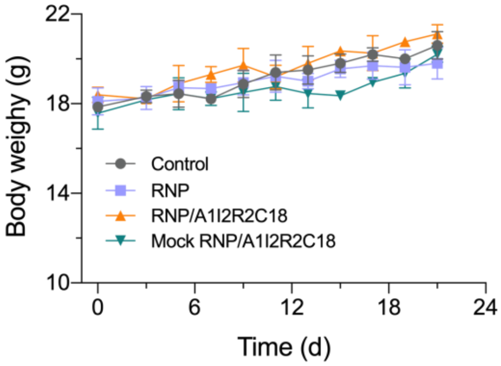

Figure S29. Body weight changes of nude mice during the experiment (n = 6).

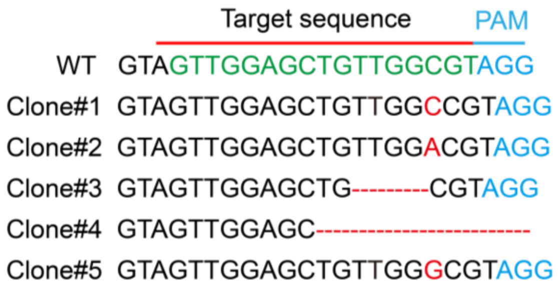

Figure S30. Sanger sequence performed by T-A cloning at *KRAS* locus from nude mice tumor. Five sequences of clones with mutations were represented.

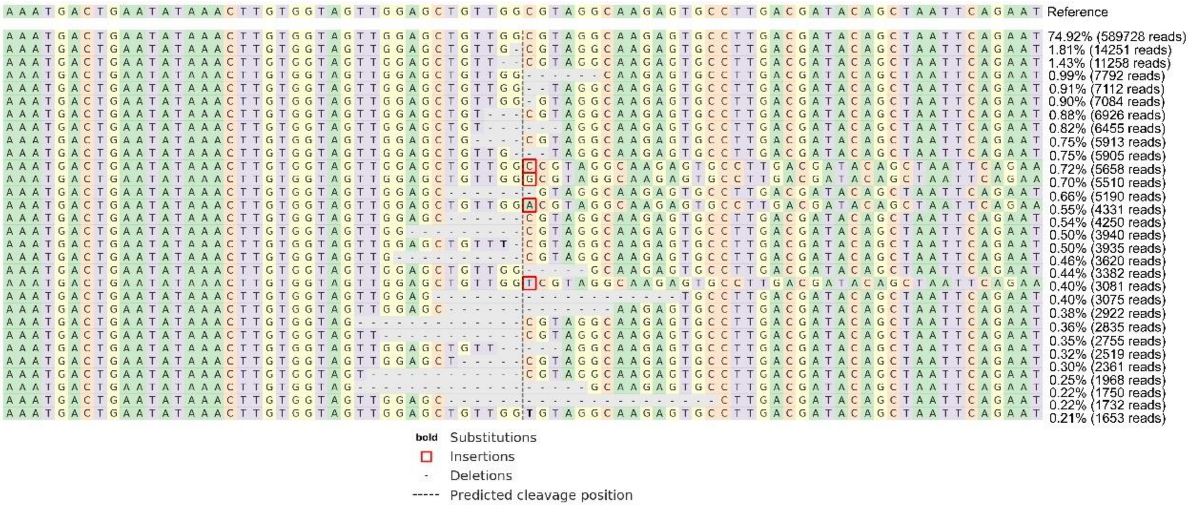

Figure S31. Deep sequencing analysis of the frequency of mutant KRAS in vivo. The mutation frequency was analyzed by a single deep sequencing library prepared from genomic DNA pooled from different groups of treated nude mice tumors.

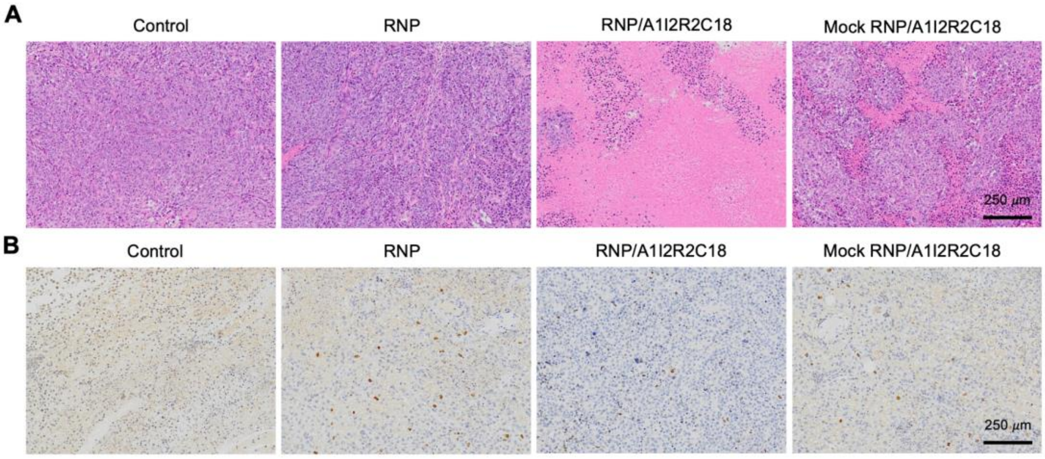

Figure S32. H&E-stained section (A) and Ki-67 immunohistochemical section (B) of the tumor tissue from the nude mice after different groups mediated treatment. Scale bar is 250 μm.

Table S1. Particle diameter and ζ-potential of various protein/GA complexes.

Protein/GA complexes	Size (nm)	ζ-potential (mV)
GFP/A1I2-1R2C18	516 ± 57	1.5 ± 0.4
SOD/A1I2-1R2C18	475 ± 10	9.1 ± 0.6
OVA/A1I2-1R2C18	671 ± 59	9.3 ± 0.4
R-PE/A1I2-1R2C18	677 ± 103	19.7 ± 1.2
Cyt-C/A1I2-1R2C18	262 ± 47	11.8 ± 2.2
Lysozyme/A1I2-1R2C18	470 ± 84	12.1 ± 3.4

Table S2. Sequences of sgRNA used in this study.

Nucleic Acid ID	Sequences (5'-3')	Notes
sgRNA-F (sgAAVS1-F)	GAAATTAATACGACTCACTATAGGCTCCCTCCCA GGATCCTCTCGTTTTAGAGCTAGAAATAGCA	T7 Promoter *AAVS1* targeting
sgRNA-R (sgAAVS1-R)	AAAAGCACCGACTCGGTGCCACTTTTTCAAGTTG ATAA CGGACTAGCCTTATTTTAACTTGCTATTTCTAGCT CTAA AAC	
sgRNA-F (sgHBB-F)	GAAATTAATACGACTCACTATAGGGTAACGGCA GACTTCTCCTCGTTTTAGAGCTAGAAATAGCA	T7 Promoter *HBB* Targeting
sgRNA-R (sgHBB-R)	AAAAGCACCGACTCGGTGCCACTTTTTCAAGTTG ATAA CGGACTAGCCTTATTTTAACTTGCTATTTCTAGCT CTAA AAC	

sgRNA-F<br>(sgEGFP-F)	GAAATTAATACGACTCACTATAGGTGAACCGCAT CGAGCTGAAGTTTTAGAGCTAGAAATAGC	T7 Promoter<br>EGFP Targeting
sgRNA-R<br>(sgEGFP-R)	AAAAGCACCGACTCGGTGCCACTTTTTCAAGTTG ATAA<br>CGGACTAGCCTTATTTTAACTTGCTATTTCTAGCT CTAA<br>AAC	
sgRNA-F<br>(sgKRAS-F)	GAAATTAATACGACTCACTATAGTTGGAGCTGTT GGCGTGTTTTAGAGCTAGAAATAGC	T7 Promoter<br>*KRAS* Targeting
sgRNA-R<br>(sgKRAS-R)	AAAAGCACCGACTCGGTGCCACTTTTTCAAGTTG ATAA<br>CGGACTAGCCTTATTTTAACTTGCTATTTCTAGCT CTAA<br>AAC	

Table S3. Primer sequences for PCR amplification of target genes.

Nucleic Acid ID	Sequences (5’-3’)
AAVS1-F	CTATGTCCACTTCAGGACAGCATGT
AAVS1-R	CCTCTTGGGAAGTGTAAGGAAGCTG
HBB-F	AACTCCTAAGCCAGTGCCAGAAGAG
HBB-R	CAGGCCATCACTAAAGGCACCGAGC
EGFP-F	ATGGTGAGCAAGGGCGAG
EGFP-R	TTACTTGTACAGCTCGTCCATGC
KRAS-F	GAGGTGGGGGTCCACTAGGA
KRAS-R	TGGACCCTGACATACTCCCAAGG

Table S4. Primers used for deep sequencing analysis.

Nucleic Acid ID	Sequences (5'-3')
KRAS-F	GGTACTGGTGGAGTATTTGATAGTG
KRAS-R	GGTCCTGCACCAGTAATATGC
