# Supplementary material for: Direct Cytosolic Delivery of Proteins and CRISPR-Cas9 Genome Editing by Gemini Amphiphiles via Non-Endocytic Translocation Pathways
Source: ACS Cent Sci. 2023 Jun 8;9(7):1313–26. doi: 10.1021/acscentsci.3c00207 (PMC10375873; doi:10.1021/acscentsci.3c00207)
Supplement: Supplementary file 2 — oc3c00207_si_002.pdf [file oc3c00207_si_002.pdf]

oc-2023-00207d.R1

Name: Peer Review Information for "Direct Cytosolic Delivery of Proteins and CRISPR-Cas9 Genome Editing by Gemini Amphiphiles via Non-Endocytic Translocation Pathways"

First Round of Reviewer Comments

Reviewer: 1

Comments to the Author

The authors introduced a facile Ugi four-component reaction to establish a molecular library of well-defined gemini amphiphiles (GA) for screening efficient cytosolic protein delivery carriers. It is interesting that the simple and single-component GA carrier was capable of delivering a broad spectrum of proteins with various molecular weights or pIs into the cytosol. The authors demonstrated that the delivery mechanism mainly depended on the lipid raft-dependent membrane fusion pathway, bypassing the conventional endocytic pathway and endosomal escape. The discovered GA carrier enables robust cytosolic delivery of hard-to-deliver Cas9 RNP for genome editing in vitro and in vivo. Of note, the GA outperformed the commercial protein delivery reagent PULSin and Cas9 RNP delivery reagent CMAX, making it a potential candidate for wide applications. Overall, this study is fascinating and meaningful, but the English writing and data explanation need to further improve. I recommend accepting it as a minor revision.

Manuscript: oc-2023-00207d

Title: Direct Cytosolic Delivery of Proteins and CRISPR-Cas9 Genome Editing by Gemini Amphiphiles via Non-Endocytic Translocation Pathways

Journal: ACS Central Science

Recommendation: Minor revision

Comments to Authors:

In this work, the authors introduced a facile Ugi four-component reaction to establish a molecular library of well-defined gemini amphiphiles (GA) for screening efficient cytosolic protein delivery carriers. It is interesting that the simple and single-component GA carrier was capable of delivering a broad spectrum of proteins with various molecular weights or pIs into the cytosol. The authors demonstrated that the delivery mechanism was mainly dependent on the lipid raft-dependent membrane fusion pathway, bypassing the conventional endocytic pathway and endosomal escape. The discovered GA carrier enable robust cytosolic delivery of hard-to-deliver Cas9 RNP for genome editing in vitro and in vivo. Of note, the GA outperformed the commercial protein delivery reagent PULSin and Cas9 RNP delivery reagent CMAX, making it as a potential candidate for wide applications. Overall, this study is very interesting and meaningful, but the English writing and data explanation need to further improve. I recommend accepting it as minor revision.

Major Comments:

1. The authors investigated GA dose-dependent cellular uptake after HeLa cells were treated with various BSA-FITC/GA complexes. How about the protein concentration-dependent cellular uptake?

2. The authors compared the effect of saturation degree of GA hydrophobic tails on protein delivery efficiency (in Figure 3F), and concluded GAs with unsaturated tails impeded cellular internalization owing to the weak affinity of the unsaturated alkyl toward the lipid raft domains. More data should be given to support this view, whether the parameters, such as particle size and protein loading efficiency, affect the cellular uptake efficiency.

3. The authors showed that GAs are capable of delivering either negatively-charged proteins or positively-charged proteins, regardless of size and pIs of proteins. Why GAs are capable of delivering positively-charged proteins? The molecular interaction between proteins and carriers should be discussed in more details.

4. In Figure 4A, the corresponding particle size and zeta potentials of various protein/GA complexes should be provided.

5. Calcein and acridine orange staining assays are mentioned in line 29 page 8, but the experiment method did not be described in the section Materials and Methods. Please supplement the details here.

6. The authors need to give all concentration units as ug/ml rather than ug/well. In Figures 2B to 2K, please clarify why a different dose of BSA/GAs was used for each characterization, and why the cytotoxicity of BSA/GAs was tested with half the amount of uptake dose.

7. To determine the cytosolic protein delivery ability of GAs, the authors used BSA-FITC protein and evaluated the fluorescence from the transfected cells. As BSA-FITC/GAs complexes may stick on the cell surface (not in the cells), the authors should quench the BSA-FITC before detecting the fluorescence.

Minor comments:

- Please accept all the change track marker in the SI document.
- Fig.S2, the author needs to clarify which cells were used here
- Please give names in a unique format for ‘BSA/A1I2-1R2C18, BSA/A1I2R2C18 and BSA/A1I2-3R2C18’.
- Please indicate cell membrane and GAs in different color.
- Page 12, line 6, please add full name of ‘PAM’
- Please add the method of ‘deep sequencing’ for the pooled DNA from SW-480
- Method of ‘Protein/GA complexes formation and characterization.’, please clarify the protein used here
- Method of ‘T7 Endonuclease I (T7E1) assay’, please add TA cloning method

Reviewer: 2

Comments to the Author

This paper describes a novel approach, gemini amphiphiles, for intracellular delivery of proteins, which adds to the current literature on amphiphile-based delivery systems. The investigators generated a library of amphiphiles with variants of each component (aldehyde, isocyanide, amine and carboxylic acid groups) and identified the chemical characteristics that are associated with the greatest cargo uptake efficiency. Proteins with MW up to 240 kDa were successfully delivered into a range of cell types (malignant and benign, murine and human, primary cells and established cell lines), and data indicating that the mode of cell entry was via lipid rafts rather than endocytosis are presented. The delivery of CRISPR/Cas9 RNP to an in vivo tumor, with subsequent introduced mutations in the target gene, KRAS, is promising.

My main comment is that, as with any platform that purports to effect intracellular delivery of biomacromolecules, the very significant challenge of how to control cell-type specific uptake following systemic administration remains. While the data shown in Fig. 5, indicate that effective cell uptake of RNP and subsequent editing of the KRAS gene occurred in vivo, this was after intratumoral delivery. Therefore, it is important that the authors comment, in the Discussion, on the issues of cell type-selectivity and the problems that are likely to occur with systemic administration.

Specific comments were as follows:

• In Fig. 2E (bright field data) the morphology of the cells exposed to the amphiphiles does seem to change (more rounded), especially post exposure to variants with longer alkyl tails. Perhaps this suggests cytotoxicity, although that would be at odds with the MTT data presented in Fig. 2C. How does the concentration of GA (1 μg/well) that was used in the MTT assay (Fig. 2C) relate to what might be used in vivo? Have the authors looked at a range of concentrations of the GAs in cytotoxicity assays – to determine the IC50 of these agents? This would be useful additional data. I would suggest presenting the concentration of the GAs as μg/mL rather than μg/well.

• The variant GA with 3 ether bonds in the spacer performs less well than those with one or two ether bonds. Do the authors have a possible explanation for this observation?

• The timing of uptake of BSA-FITC seems to peak at ca 4 h in Fig.3 B/C and yet it is shown to be much quicker (90 s) in Fig. 3G, both in HeLA cells. How do the authors explain this apparent inconsistency?

• In Fig.4 A – it might be better to present the small bar charts (MFI fold change) with the same scale on the y-axis (so comparisons between proteins are easier).

• Fig. 4F – I wasn't clear about the function of heparin in this experiment. Could a little more explanation be added to the text.

• Fig. 5C/D: "Maker" is misspelled. The marker sizes should be added.

Author's Response to Peer Review Comments:

April 20, 2023

Dear editor,

Thanks for your letter concerning our manuscript entitled "**Direct Cytosolic Delivery of Proteins and CRISPR-Cas9 Genome Editing by Gemini Amphiphiles via Non-Endocytic Translocation Pathways**" (Manuscript ID: oc-2023-00207d). We would like to thank you for giving us the opportunity to revise this manuscript, and we also greatly appreciate the editors and reviewers for their constructive comments to improve our manuscript. The manuscript has been revised according to their comments, all changes made are highlighted in red in the revised manuscript, and our point-by-point responses are given below. We strongly believe that these critiques and responses have helped to improve the quality of our manuscript. We hope now that the revised manuscript can be accepted for publication in ***ACS Central Science***.

Thank you again and we look forward to your favorable consideration.

Yours sincerely,

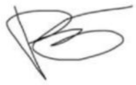

Yuan Ping, PhD
Professor, Institute of Pharmaceutics,
College of Pharmaceutical Sciences,
Zhejiang University, Hangzhou, 310058, China
E-mail: pingy@zju.edu.cn
Tel: 86-198-1786-5446

Response to the Comments of Editors and Reviewers

The Editor's Comments:

1. SYNOPSIS: ACS Central Science requires a brief synopsis. The synopsis should be no more than 200 characters (including spaces) and should reasonably correlate with the Table of Contents (TOC) graphic. The synopsis is intended to explain the importance of the article to a broader readership across the sciences. Please place your synopsis in the manuscript file after the TOC graphic.

Response: Thank you. The synopsis was provided in this revision as below:

"A robust gemini amphiphile-based carrier platform for facilitating direct cytosolic delivery of broad-spectrum proteins, including Cas9 ribonucleoproteins, through the lipid raft-dependent membrane fusion mechanism." (**on page 36**)

2. SI FILE: Please ensure that tracked changes is turned off in your Supporting Information file.

Response: Thank you. We have revised it accordingly.

Reviewer: 1

In this work, the authors introduced a facile Ugi four-component reaction to establish a molecular library of well-defined gemini amphiphiles (GA) for screening efficient cytosolic protein delivery carriers. It is interesting that the simple and single-component GA carrier was capable of delivering a broad spectrum of proteins with various molecular weights or pIs into the cytosol. The authors demonstrated that the delivery mechanism mainly depended on the lipid raft-dependent membrane fusion pathway, bypassing the conventional endocytic pathway and endosomal escape. The discovered GA carrier enables robust cytosolic delivery of hard-to-deliver Cas9 RNP for genome editing in vitro and in vivo. Of note, the GA outperformed the commercial protein delivery reagent PULSin and Cas9 RNP delivery reagent CMAX, making it a potential candidate for wide applications. Overall, this study is fascinating and meaningful, but the English writing and data explanation need to further improve. I recommend accepting

it as a minor revision.

Response: We greatly appreciate the reviewer for positive feedback on our work. In this revision, the English writing and data explanation have been further improved accordingly.

Major comments:

1. The authors investigated GA dose-dependent cellular uptake after HeLa cells were treated with various BSA-FITC/GA complexes. How about the protein concentration-dependent cellular uptake?

Response: Thanks for raising this concern. In this revision, we supplemented the data on BSA-FITC concentration-dependent cellular uptake in the **Supporting Information**. As shown in **Figure S12**, the BSA-FITC delivered by the representative A1I2R2C18 showed increased fluorescence intensity in a protein dose-dependent manner.

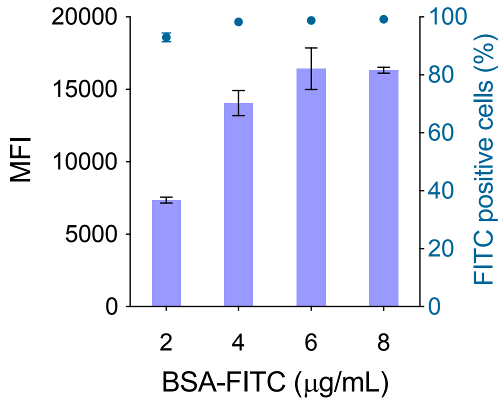

Figure S12. MFI and BSA-FITC positive cells after HeLa cells incubated with BSA-FITC/A1I2R2C18 complexes at varied BSA-FITC concentrations for 4 h. A1I2R2C18 was kept constant at 8 μg/mL (n = 3).

2. The authors compared the effect of saturation degree of GA hydrophobic tails on protein delivery efficiency (in Figure 3F), and concluded GAs with unsaturated tails impeded cellular internalization owing to the weak affinity of the unsaturated alkyl toward the lipid raft domains. More data should be given to support this view, whether the parameters, such as particle size and protein loading efficiency, affect the cellular uptake efficiency.

Response: Thanks for raising this suggestion. In this revision, we further provided the particle

size, ζ-potential, and BSA loading efficiency of BSA/A1I2R2C18-1 and BSA/A1I2R2C18-2 complexes, respectively. These data have been added in the **Supporting Information**, **Figure S15,** and the following discussions have been added in the revised manuscript (**on page 9**).

"We found that the corresponding BSA/A1I2R2C18-1 and BSA/A1I2R2C18-2 complexes showed very similar particle size, surface positive charge and BSA loading efficiency (> 90%), as compared to the BSA/A1I2R2C18 (**Figure S15**)."

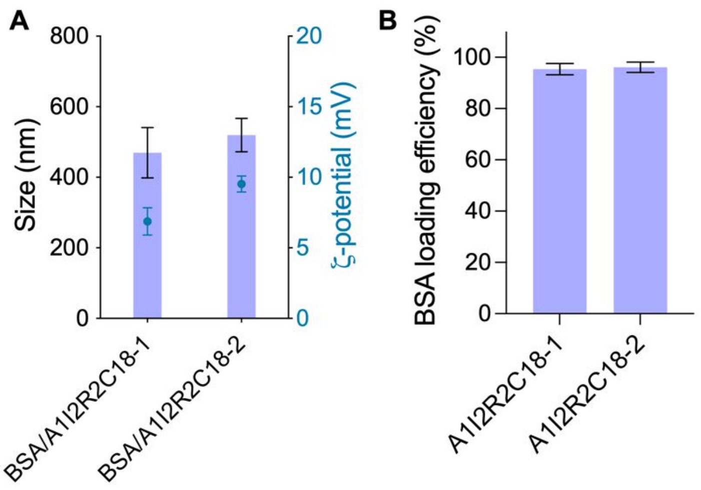

Figure S15. A) Particle size and ζ-potential of BSA/A1I2R2C18-1 and BSA/A1I2R2C18-2 complexes (n = 3). B) Protein loading efficiency of BSA/A1I2R2C18-1 and BSA/A1I2R2C18-2 complexes (n = 4). BSA was 4 μg/mL, and A1I2R2C18-1 and A1I2R2C18-2 were 8 μg/mL, respectively.

3. The authors showed that GAs are capable of delivering either negatively-charged proteins or positively-charged proteins, regardless of size and pIs of proteins. Why GAs are capable of delivering positively-charged proteins? The molecular interaction between proteins and carriers should be discussed in more details.

Response: Thanks for raising this point. More data and discussions have been added in the revised manuscript (**on page 10**).

"Generally, protein surfaces are chemically heterogeneous and contain cationic, anionic and hydrophobic amino acid residues, hence they could co-assemble with the GAs to form complexes through hydrophobic force and/or electrostatic interactions (**Table S1**)"

4. In Figure 4A, the corresponding particle size and zeta potentials of various protein/GA complexes should be provided.

Response: Thanks for this suggestion. In this revision, we added the corresponding particle size and ζ-potential of various protein/GA complexes in the **Supporting Information, Table S1** as below:

Table S1. Particle diameter and ζ-potential of various protein/GA complexes

Protein/GA complexes	Size (nm)	ζ-potential (mV)
GFP/A1I2-1R2C18	516 ± 57	1.5 ± 0.4
SOD/A1I2-1R2C18	475 ± 10	9.1 ± 0.6
OVA/A1I2-1R2C18	671 ± 59	9.3 ± 0.4
R-PE/A1I2-1R2C18	677 ± 103	19.7 ± 1.2
Cyt-C/A1I2-1R2C18	262 ± 47	11.8 ± 2.2
Lysozyme/A1I2-1R2C18	470 ± 84	12.1 ± 3.4

5. Calcein and acridine orange staining assays are mentioned in line 29 page 8, but the experiment method did not be described in the section Materials and Methods. Please supplement the details here.

Response: Thanks for raising this point. The methods including calcein assay and acridine orange assay have been supplemented in the revised manuscript, as seen in the section MATERIALS AND METHODS (**on page 19**).

"**Calcein assay.** HeLa cells were seeded into glass-bottom dishes and incubated overnight. After being washed with PBS, the cells were treated with DMEM containing 150 μg/mL of calcein with or without BSA/A1I2R2C18 complexes. After incubating for 4 h, the cells were washed with PBS to remove extracellular calcein, and then viewed under the CLSM.

Acridine orange assay. HeLa cells were seeded into 24-well plates and incubated overnight. After being incubated with DMEM alone, BSA alone or BSA/A1I2R2C18 complexes for 4 h, the cell medium was removed, and cells were incubated with 2.5 μg/mL of acridine orange

solution for another 15 min. The endosomal/lysosomal membrane permeability was tested by flow cytometry with the excitation at 488 nm, and the emission at 530 (green fluorescence) or 620 nm (red fluorescence), respectively."

6. The authors need to give all concentration units as μg/ml rather than μg/well. In Figures 2B to 2K, please clarify why a different dose of BSA/GAs was used for each characterization, and why the cytotoxicity of BSA/GAs was tested with half the amount of uptake dose.

Response: Thanks for raising this concern. The concentrations of BSA or BSA-FITC were 4 μg/mL and GAs were 8 μg/mL, and they were kept the same in **Figure 2B–K**. In fact, the dose units are dependent on the volume of cell culture medium added in each well, for example, in **Figure 2D**, we added 0.5 mL of culture medium containing 4 μg/mL of BSA-FITC and 8 μg/mL of GAs, so the calculated dose units are 2 μg/well for BSA-FITC and 4 μg/well for GAs. Similarly, the dose units are 0.5 μg/well for BSA and 1 μg/well for GAs as calculated from 0.125 mL of culture medium containing 4 μg/mL of BSA and 8 μg/mL of GAs in an MTT assay (**Figure 2C**). In this revision, to eliminate misunderstanding by readers, as suggested by the reviewer, the data presentations by changing from the dose unit "μg/well" to the concentration unit "μg/mL" have been given in the revised manuscript.

7. To determine the cytosolic protein delivery ability of GAs, the authors used BSA-FITC protein and evaluated the fluorescence from the transfected cells. As BSA-FITC/GAs complexes may stick on the cell surface (not in the cells), the authors should quench the BSA-FITC before detecting the fluorescence.

Response: Thanks for raising this point. In this revision, we supplemented the comparison data before and after trypan blue quenching. As shown in **Figure S13, Supporting Information**, we observed that the fluorescence intensity of transfected HeLa cells has no change before and after treatment of trypan blue, which is a membrane-impermeable dye to quench the fluorescence of BSA-FITC absorbed on cell membranes, suggesting that the delivered BSA-FITC was almost distributed inside cells. These discussions have been added in the revised manuscript (**on page 8**)

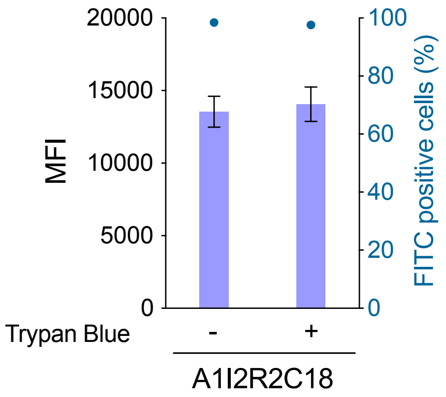

Figure S13. MFI and BSA-FITC positive cells of BSA-FITC/A1I2R2C18-transfected HeLa cells before and after trypan blue quenching. Trypan blue was 0.4 mg/mL, BSA-FITC was 4 μg/mL and A1I2R2C18 was 8 μg/mL (n = 3).

Minor Comments:

1. Please accept all the change track marker in the SI document.

Response: Thank you. We have accepted all the change track markers in the revised Supporting Information.

2. Fig.S2, the author needs to clarify which cells were used here.

Response: Thank you. **Figure S2** was an extended data analysis of **Figure 1C**, and the experiment was performed on HeLa cells. In this revision, this has been clarified as seen in the legend of **Figure S2**.

3. Please give names in a unique format for 'BSA/A1I2-1R2C18, BSA/A1I2R2C18 and BSA/A1I2-3R2C18'.

Response: Thanks for your suggestion. In this study, more than 150 GAs and 10 different types of proteins were investigated, therefore we suggested adopting the same format of "protein/GA" to clearly indicate what exact proteins and GAs contributed to the formed complexes. Using other abbreviations or unique formats may cause unwanted confusion to readers.

4. Please indicate cell membrane and GAs in different color.

Response: Thank you. We adjusted the color of cell membranes (**Figure 3H**) in the revised

manuscript (**on page 31**)

5. Page 12, line 6, please add full name of 'PAM'

Response: Thank you. We have added the full name of 'PAM' on page 12.

"protospacer adjacent motif (PAM)"

6. Please add the method of 'deep sequencing' for the pooled DNA from SW-480

Response: Thanks for raising this point. In this revision, we added "Deep sequencing assay" in the section MATERIALS AND METHODS (**on page 22**).

"**Deep sequencing assay**. A deep sequencing assay was conducted using the following procedure. Evaluation and design of off-target loci were performed using the CasOFFinder website (www.rgenome.net/cas-offinder/). Amplification of corresponding fragments was accomplished by utilizing specific primers (**Table S4**), referred to as the first PCR product. Following the amplification of corresponding fragments designed by the CasOFFinder website, PCR/Gel Extraction and Purification kits (Vazyme Biotech Co., Ltd) were utilized to purify the resulting product. The purified product was subsequently subjected to further amplification to generate PCR fragments with a size limit of 250 base pairs (bp), encompassing the target gene loci, and referred to as the second PCR product. The extracted and purified product from the previous step underwent further amplification using primers containing an index sequence. The resulting product was again purified using PCR/Gel Extraction and Purification kits. Finally, the products underwent sequencing analysis, and the obtained data were analyzed using CRISPResso2 software based on the provided instructions."

7. Method of 'Protein/GA complexes formation and characterization.', please clarify the protein used here.

Response: Thank you. We have clarified the used proteins in the method of 'Protein/GA complexes formation and characterization' (**on page 17**).

"The protein solutions including BSA, GFP, SOD, OVA, R-PE, Cyt-C, lysozyme, saporin, β-Gal, and Cas9 RNP were also prepared in Hepes buffers."

8. Method of 'T7 Endonuclease I (T7E1) assay', please add TA cloning method.

Response: Thank you. In this revision, we have supplemented the "T-A cloning and Sanger sequencing" in the section MATERIALS AND METHODS (**on page 21**).

"To perform T-A cloning and Sanger sequencing, we followed the protocol provided by TSINGKE Co., Ltd. Specifically, the amplified DNA fragment was cloned into the T vector and subsequently transformed into` DH5α. Monoclonal was selected and sent for Sanger sequencing at Youkang Biotech Co., Ltd. The obtained DNA sequences were aligned with the target-gene locus using SnapGene software for analysis."

Reviewer: 2

This paper describes a novel approach, gemini amphiphiles, for intracellular delivery of proteins, which adds to the current literature on amphiphile-based delivery systems. The investigators generated a library of amphiphiles with variants of each component (aldehyde, isocyanide, amine and carboxylic acid groups) and identified the chemical characteristics that are associated with the greatest cargo uptake efficiency. Proteins with MW up to 240 kDa were successfully delivered into a range of cell types (malignant and benign, murine and human, primary cells and established cell lines), and data indicating that the mode of cell entry was via lipid rafts rather than endocytosis are presented. The delivery of CRISPR/Cas9 RNP to an in vivo tumor, with subsequent introduced mutations in the target gene, KRAS, is promising.

Response: We greatly appreciate the reviewer for positive feedback on our work and the comments to improve our manuscript.

1. My main comment is that, as with any platform that purports to effect intracellular delivery of biomacromolecules, the very significant challenge of how to control cell-type specific uptake following systemic administration remains. While the data shown in Fig. 5, indicate that effective cell uptake of RNP and subsequent editing of the KRAS gene occurred in vivo, this was after intratumoral delivery. Therefore, it is important that the authors comment, in the Discussion, on the issues of cell type-selectivity and the problems that are likely to occur with

systemic administration.

Response: Thanks for your professional and constructive comments. In this revision, discussions on the issue of cell type-selectivity have been added as suggested in the section DISCUSSION (**on page 14**).

"Despite these promising results, it is unclear whether GAs are stable and efficient in vivo after systemic administration, and the issue of cell-type selectivity after systemic administration needs to be solved to reduce off-target toxicity, hence their safety in vivo remains elusive despite their lower cytotoxicity in vitro. Therefore, future work will be dedicated to understanding the influence of surface functionalization on cell-type specific uptake and their safety, efficacy, and biodistribution in vivo"

2. In Fig. 2E (bright field data) the morphology of the cells exposed to the amphiphiles does seem to change (more rounded), especially post exposure to variants with longer alkyl tails. Perhaps this suggests cytotoxicity, although that would be at odds with the MTT data presented in Fig. 2C. How does the concentration of GA (1 μg/well) that was used in the MTT assay (Fig. 2C) relate to what might be used in vivo? Have the authors looked at a range of concentrations of the GAs in cytotoxicity assays – to determine the IC50 of these agents? This would be useful additional data. I would suggest presenting the concentration of the GAs as μg/mL rather than μg/well.

Response: Thanks for raising these concerns. The bright field data in **Figure 2E** were captured after HeLa cells were incubated with various BSA-FITC/GA complexes for 4 h and then washed with PBS but without fixed treatment, so the cell morphology may slightly change due to long time of exposure to room temperature during image capture. As seen in **Figure 3B**, the morphology of cells treated with BSA-FITC/A1I2R2C18 complexes is normal after various incubation time, because of the fixed treatment before image capture. Besides, as the additional evidence from the afforded bright field as below, after HeLa cells incubated with BSA-FITC/A1I2R2C18 for 4 h, we instantly captured cell image before PBS washing (B), and they kept the same morphology as cells without any treatment (A), indicating negligible cytotoxicity after treatment with BSA-FITC/A1I2R2C18 complexes. This is highly consistent with the result of MTT assay (**Figure 2C**).

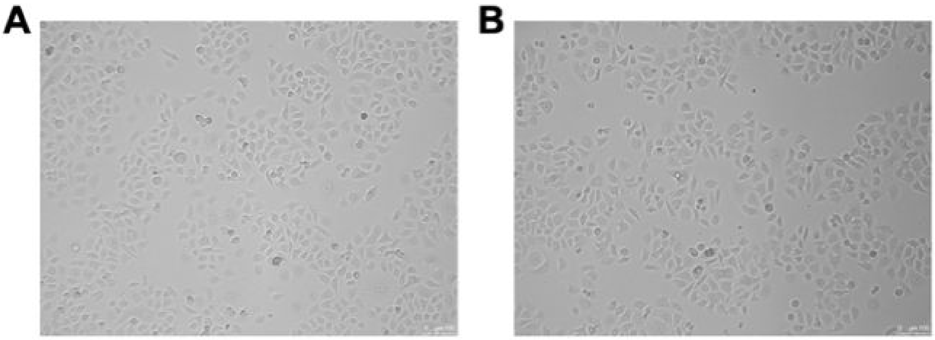

Figure legend: (**A**) Bright field image of HeLa cells without any treatment. (**B**) Bright field image of HeLa cells after incubation with BSA-FITC/A1I2R2C18 complexes for 4 h. These cell images were instantly captured before PBS washing.

In the MTT assay, the dose unit of 1 μg/well corresponded to the concentration unit of 8 μg/mL of GAs. As suggested by the reviewer, we have changed all dose units "μg/well" to the concentration units "μg/mL" in this revised manuscript. We further supplemented the dose-dependent cytotoxicity of GAs and their corresponding 50% inhibitory concentration (IC50) values as shown in **Figure S4, Supporting Information**, the result demonstrated that the cytocompatibility of GAs could be greatly improved by increasing the alkyl chain length from C16 to C20 due to the higher 50% inhibitory concentration (IC50) values. For intracellular delivery of Cas9 RNP for CRISPR-Cas9 genome editing, the dosage of GAs used in vivo was dependent on the dosage of Cas9 protein (RNP). To enable robust genome editing efficiency, we confirmed that the mass ratio of 1: 2 is optimized for Cas9 protein and A1I2R2C18 (**Figure S24**). To improve the antitumor efficacy in vivo, we used 1 mg/kg of Cas9 protein and the corresponding A1I2R2C18 was 2 mg/kg here. As evidenced that Mock RNA/A1I2R2C18 has no significant tumor inhibition (**Figure 5H and Figure S28**) and body weights of the treated mice have no obvious change as compared to the control (**Figure S29**), which suggested that the used dosage of A1I2R2C18 in vivo was safe through peritumor administration.

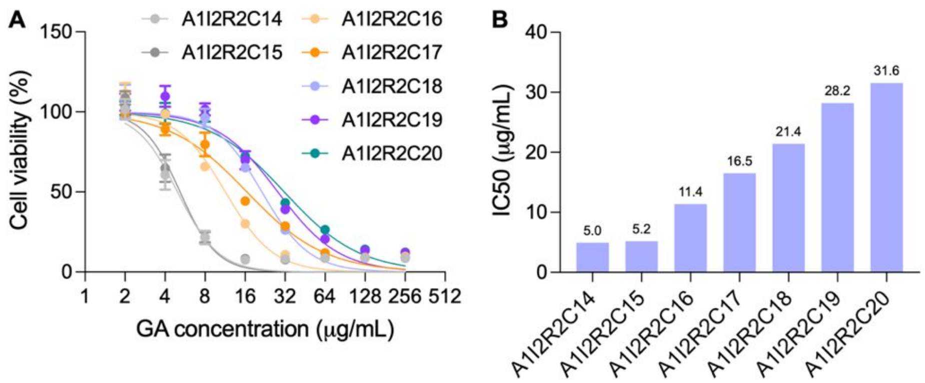

Figure S4. GA-dose dependent relative cell viability (A) and the corresponding 50% inhibitory concentration (IC50) values of each GA (B) (n = 4).

3. The variant GA with 3 ether bonds in the spacer performs less well than those with one or two ether bonds. Do the authors have a possible explanation for this observation?

Response: Thanks for raising this point. It was reported that a longer hydrophilic spacer can increase the overall hydrophilicity of GAs (*Zana, R. Dimeric (Gemini) Surfactants: Effect of the Spacer Group on the Association Behavior in Aqueous Solution. J. Colloid Interface Sci. 2002, 248, 203–220*). Here, we measured the critical aggregation concentration (CAC) of A1I2-3R2C18 with 3.5- and 3.3-fold higher than A1I2-1R2C18 and A1I2R2C18, respectively (**Figure S11, Supporting Information**), which suggested that the decreased hydrophobicity of A1I2-3R2C18 likely leads to the negative effect on its affinity to lipophilic cell membranes and therefore decrease intracellular protein delivery efficiency. The data and discussions have been added in this revised manuscript (**on page 7**).

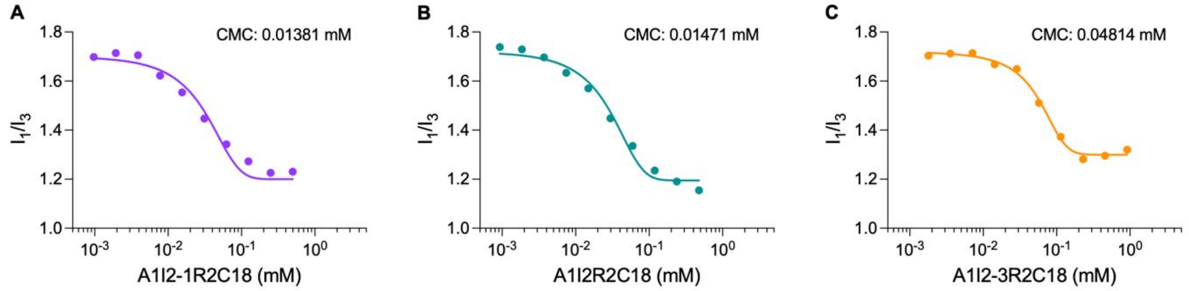

Figure S11. Critical aggregation concentration (CAC) of A1I2-1R2C18 (A), A1I2R2C18 (B), and A1I2-3R2C18 (C) as determined by the pyrene probe method.

4. The timing of uptake of BSA-FITC seems to peak at ca 4 h in Fig.3 B/C and yet it is shown

to be much quicker (90 s) in Fig. 3G, both in HeLa cells. How do the authors explain this apparent inconsistency?

Response: Thanks for raising this concern. Fluorescence images in **Figure 3B, C** presented the **accumulative cellular uptake** of BSA-FITC/A1I2R2C18 complexes and had a time-dependent increase in intracellular fluorescence intensity after 0.5–4 h incubation. While, for the individual cell, we noticed that **the entry event** starts as early as 0.5 h incubation. Although the FITC-positive percentage of cells is low at initial stage, as the increase of incubation time, cells could uptake more BSA-FITC/A1I2R2C18 complexes and reach a plateau at 4 h. Of note, In **Figure 3G**, we aimed to monitor the **transient intracellular delivery event** of BSA-FITC/A1I2R2C18 complexes after cellular uptake, through the time-lapse imaging when HeLa cells were incubated with complexes for 1 h and recorded the image in a 30-second interval. The result indicated that the green fluorescence of delivered BSA-FITC thoroughly spreading all over the cytosol could be achieved within 90 s when the individual cell ingested complexes.

5. In Fig.4 A – it might be better to present the small bar charts (MFI fold change) with the same scale on the y-axis (so comparisons between proteins are easier).

Response: Thanks for your suggestion. In this revision, all y-axis scales were set at 0–6 in the small bar charts in **Figure 4A** (**on page 33**).

6. Fig. 4F – I wasn't clear about the function of heparin in this experiment. Could a little more explanation be added to the text.

Response: Thanks for raising this point. In this revision, more explanation has been added **on page 11** "β-Gal recovered from β-Gal/A1I2-1R2C18 complexes still maintained high enzymatic function after the addition of heparin, which is a highly sulfated glycosaminoglycan with strong negative charges that can trigger the release of native β-Gal from the β-Gal/A1I2-1R2C18 complexes by competitive binding with the cationic A1I2-1R2C18."

7. Fig. 5C/D: "Maker" is misspelled. The marker sizes should be added.

Response: Thank you. In this revision, we have added the marker size in **Figure 5C, D, and J**, and **Figure S19 and S21**.
